# Etching characteristics and profile simulation of silicon

**DOI:** 10.1371/journal.pone.0356817

**Published:** 2026-08-25

**Authors:** Hui Zhang, Pengpeng Cai

**Affiliations:** 1 Industrial Perception and Intelligent Manufacturing Equipment Engineering Research Center of Jiangsu Province, Nanjing University of Industry Technology, Nanjing, Jiangsu, China; 2 The Southeast University, Nanjing, Jiangsu, China; Swiss Federal Technology Institute of Lausanne, SWITZERLAND

## Abstract

The anisotropic wet etching characteristics of crystal silicon are complex and easily affected by etching conditions and mask shapes, which makes it difficult to accurately predict and control the evolution process and 3D etched profile. This study is based on experimental data on the etching rate and mask etching structure of crystal silicon, and analyzes in detail the anisotropic etching mechanism and mask etching forming process, and then establishes an etching process simulation model using the Level Set method. The model first obtains the etching rate of all crystal planes by interpolation of the etching rate of a few sample crystal planes, then establishes the control equation of the etching motion interface based on the level set function and uses the finite difference method to solve the equation to track the evolution of the etching interface, and finally achieves the high-precision simulation of the 3D etched profile under various masks (window mask, mesa mask and double-sided mask). Experimental results show that the simulation results of the model are in good agreement with the actual situation, and the model has good adaptability and predictive ability for different etching systems and mask wafers with different shapes. In addition, the modeling theory is also applicable to the etching profile simulation of quartz and other crystalline materials, and has high simulation accuracy.

## 1. Introduction

The anisotropic wet etching process is a processing method that utilizes the non-isotropic reaction between chemical etching solutions and the target crystal to form structures. It can produce micron-scale 3D structures (such as cantilever beams and thin-film cavities) with specific angles and smooth surfaces, which meets the fundamental requirements of mechanical performance and geometric accuracy for MEMS devices. Meanwhile, it demonstrates unique advantages of high selectivity and efficiency in critical TSV fabrication steps, including backside silicon thinning and tapered hole formation. Due to the physical constraints of crystallographic etching, liquid surface tension effects, and lateral undercut, the precision and controllability of wet etching process plummet at the nanoscale (<100 nm), failing to meet atomic-level dimensional control requirements. Consequently, its research findings are primarily confined to relatively large-scale microstructures (TSVs and MEMS), forming a highly complementary and industrially specialized relationship with nanomanufacturing technologies [1–5]. In industrial production, anisotropic etching of silicon substrates typically employs etching agents such as TMAH and KOH. The silicon substrate is then coated with a mask along specific crystal planes (e.g., [100], [110], [111]) to selectively remove excess material, achieving precise microstructure processing. The key to this process lies in the selective utilization of crystal planes with different etching rates [6–11]. Compared with laser-assisted processing, wet etching offers low-cost, large-area batch processing with simple equipment and high throughput, though its etch rate is moderate (TMAH: 0.5–1 μm/min) and feature size is micron-limited. KOH is ~ 80% cheaper and more anisotropic but causes K⁺ contamination. Laser processing provides maskless flexibility and rapid prototyping for small batches, but its cost advantage is context-dependent: equipment may exceed £100K versus ~£50K for wet etching, and operational costs are high, so wet etching remains more economical for high-volume manufacturing. To reduce trial-and-error in mask design, computational simulations with level-set methods automatically optimize masks and etch time, achieving <5% volumetric deviation from target, thus shortening development and lowering costs. Integrating laser techniques with wet etching can enhance competitiveness in micro/nano manufacturing [12–14].

For the KOH + IPA etching system (KOH, H_2_O, and (CH_3_)_2_CHOH), the silicon etching reaction equation is as follows:


$$\begin{array}{c} \left\{ \begin{array}{c} @l@\text{KOH}+{\text{H}}_{2}0\to {\text{K}}^{+}+2{\text{OH}}^{\text{-}}+{\text{H}}^{+}\\ \text{Si}+2{\text{OH}}^{\text{-}}+4{\text{H}}_{2}0\to {\text{Si(OH)}}_{\text{6}}^{2-}\\ {\text{Si(OH)}}_{\text{6}}^{2-}+6{\text{(CH}}_{3}{)}_{2}\text{CHOH}\to [{\text{Si(OC}}_{3}{\text{H}}_{7}{)}_{6}{]}^{2-}+6{\text{H}}_{2}0 \end{array}\right. \end{array}$$ (1)

In the absence of (CH_3_)_2_CHOH), the chemical reaction proceeds as follows:


$$\begin{array}{c} \text{Si}+\text{2KOH}+{\text{H}}_{2}0\to {\text{K}}_{\text{2}}{\text{SiO}}_{\text{3}}+2{\text{H}}_{2}↑ \end{array}$$ (2)

In the aforementioned etching reaction, the IPA acts as an additive to enhance surface quality and regulate the etching rate of crystal planes: 1) It reduces solution surface tension, facilitating hydrogen bubble detachment from silicon surfaces to prevent uneven etching caused by bubble adhesion, thereby improving etched surface quality. 2) It inhibits OH⁻ activity, slowing the etching reaction rate by adsorbing onto crystal planes rich in single dangling bonds, forming surface groups (≡Si-OR[$R=-CH\;(CH_{\text{3}}{)}_{\text{2}}\backslash )$]) that decelerate the etching process [15].

In fact, the anisotropic characteristics of wet etching in crystalline materials often exhibit highly complex behavior. The etched profile and reaction rates are influenced not only by the physicochemical properties of the etching solution (concentration, diffusion, and adsorption, etc.), but also by factors such as the material’s crystal orientation, mask geometry, and surface roughness. These variables collectively make the etching process and its outcomes challenging to predict and control. To clarify the anisotropic etching behavior of silicon and accurately simulate etched profile under arbitrary masks in various etching conditions, the current research primarily employs two approaches: atomic methods (e.g., Monte Carlo and Cellular Automata) and geometric methods (e.g., Wulff-Jaccodine and Level Set) [3, 16–20]. The study obtained an etching rate distribution curve with all silicon planes through hemispherical etching experiments, and first analyzed the anisotropic etching process of the window and convex mask wafers. Second, an interpolation rate model was established, which can obtain the etching rate of any plane by interpolating the etching rates of a small number of sample planes. Finally, a 3D etched profile model for silicon was constructed based on the Level-Set method. This model not only enables dynamic tracking of etching interfaces but also effectively simulates the etching structures of various types of mask wafers (concave, convex, and double-sided masks).

## 2. Experimental analysis of silicon anisotropic wet etching

### 2.1. Experiment to obtain all crystal plane etching rates

The most widely used methods for determining the etching rates of all silicon planes include the outer ring method, the vertically micromachined wagon wheel method and the hemispherical etching method, and so on [21, 22]. Among these, the hemispherical etching method provides the most comprehensive and accurate etching rate data. This study employs the hemispherical etching method, with the etching process and experimental data as follows [10, 23]:

- Etching Time: 45 minutes (Stirring: 100 r/min);
- Etching Solution: 40wt % KOH, 70°C (Range: ± 3°C);
- Etching Object: Silicon (D = 45 mm ± 0.1, Sphericity φs ≥ 0.99, Rotating axis: [100], equatorial plane:(100));
- Measuring Method:Mechanical probe (longitude α:5°,latitudeβ:5°).
- Measurement Equipment:PMM-C coordinate measuring machine (accuracy:1.5μm).

**Fig 1:**
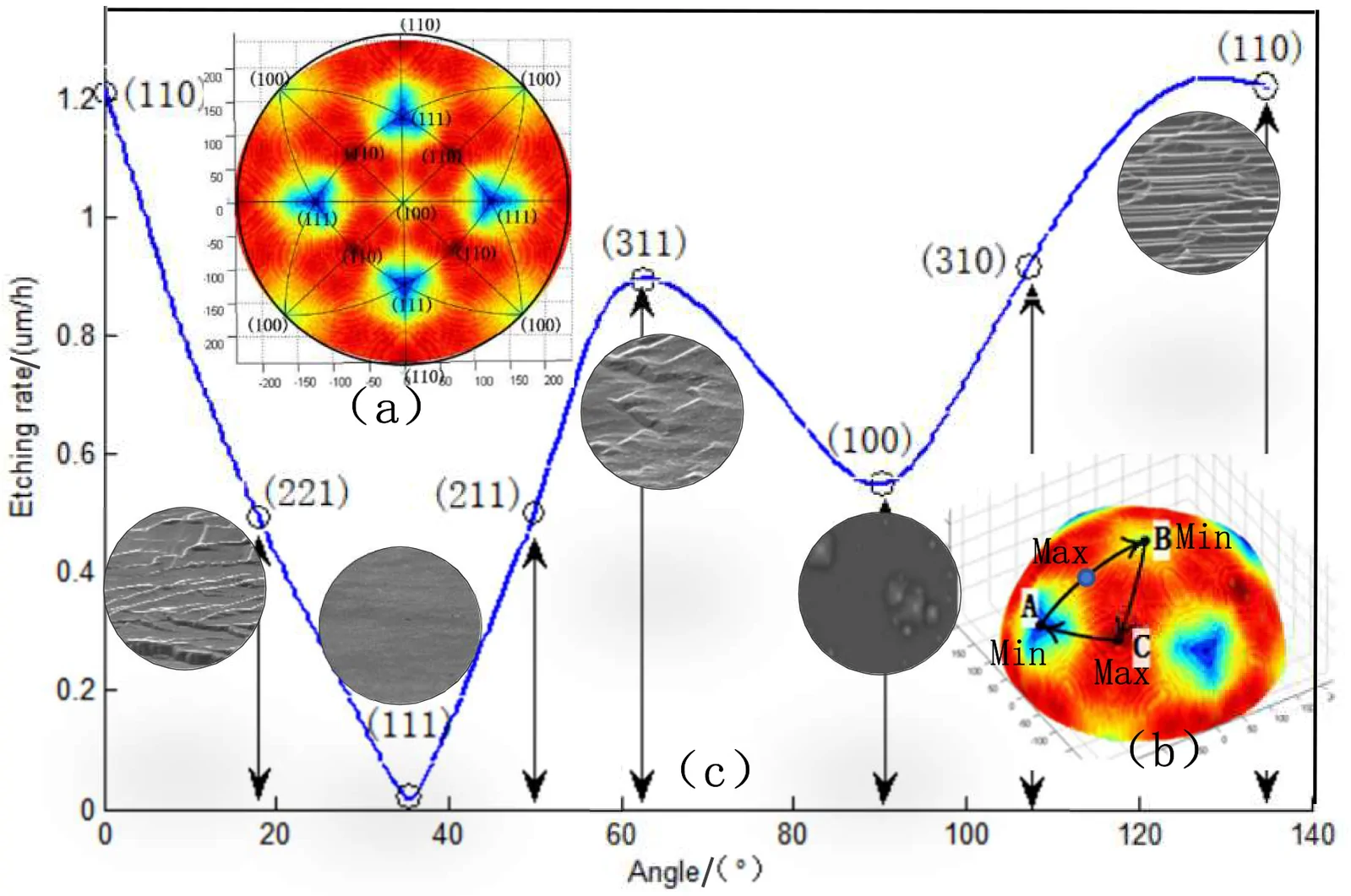
Etching experiment of silicon hemisphere: (a) A typical stereographic projection of the etching rate distribution on the sphere(red: high etching rate, blue: low etching rate); (b)The actual etching rate distribution right on the sphere. (c) The etching rate curve composed of all planes and etching surface morphology of key planes.

According to the above experimental conditions, this study obtained A typical stereographic projection of the etch rate distribution on the sphere and extracted the etching rate curve of all silicon planes, as well as the etching morphology of some key planes [24], as shown in Fig 1(in the uploaded file). Above data are consistent with the etching rate distribution obtained by Gosalvez, M A et al. using the wagon wheel method in 2011 [25], but the data from the hemispherical the etched hemisphere method are more comprehensive and relatively more accurate. The etching rate distribution of all silicon planes exhibits obvious 90°rotational symmetry in Fig 1 (a), and all planes are distributed on the spherical surface of the A-B-C line in Fig 1 (b). From point A to point B, the etching rate gradually increases and reaches the maximum rate on the crystal planes before gradually decreasing. From point B to point C, the etching rate gradually increases to its maximum value. When moving from point C to point A, the etching rate rapidly decreases from a maximum value to a minimum value [26]. Further analysis showed that the two maximum values are located at the (110) on the (100) – (110) – (100) crystallographic zones and the (311) on the (100) – (111) – (110) crystallographic zones, and V_etch (110)>V_etch (311); the two minima are mainly distributed at the (100) of the hemisphere vertex and at the (111) on the (100) – (111) – (110) crystallographic zones, and V_etch (100)>>V_etch (111)≈0. From the above, it can be concluded that the etching rates of the A-B-C connection crystal planes are the curve of the etching rates of the whole silicon crystal planes. The curve is in the shape of a “W,” with (110) being the crystal plane with the highest rate and (111) being the crystal plane with the lowest rate. Different crystal planes show significant differences in surface morphology after etching, as shown in Fig 1 (c).

### 2.2. Analysis of the Silicon Microstructure Formation Process

**Fig 2:**
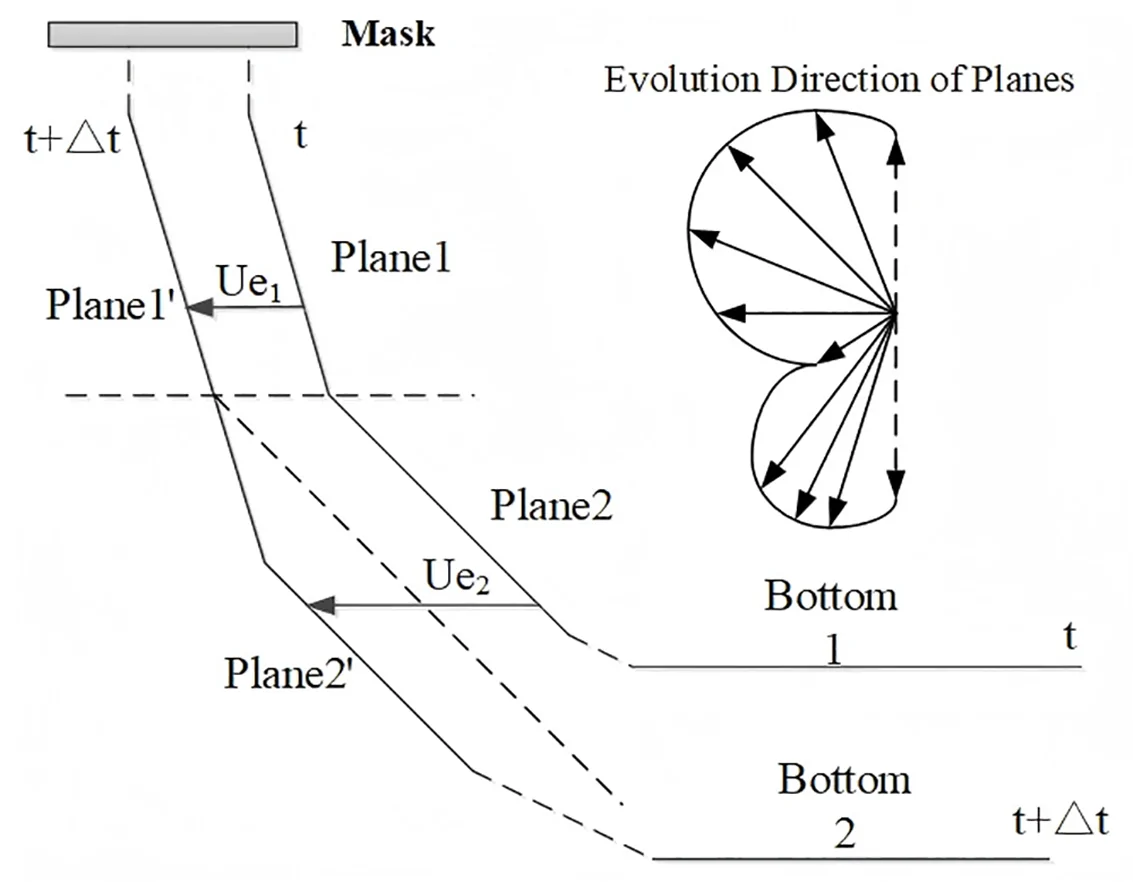
Evolution process of sidewall etching at the boundary of the mask.

The evolution and formation of silicon microstructures etched is primarily influenced by two etching orientations: the cross-sectional polygon evolution (completely vertical) and the upper contour evolution (completely horizontal) [27, 28]. According to WJ etching theory, the crystal plane with the minimum undercutting rate will gradually occupy the dominant position in the etching process and eventually form the etching profile.For the cross-sectional polygon evolution, the sidewalls of the formed structure are primarily composed of crystal planes with minimal undercutting rates parallel to the mask boundary. As shown in Fig 2 (in the uploaded file), the undercutting rates of planes Plane1 and Plane2 are denoted as Ue_1_ and Ue_2_, respectively. When Ue_1_ = Ue_2_, Plane1 and Plane2 have equivalent status during the etching process, and neither can cover the other, resulting in the simultaneous presence of both crystal planes. When Ue_1_ < Ue_2_, Plane1 becomes dominant during the etching process, causing Plane2 to gradually disappear.

**Fig 3:**
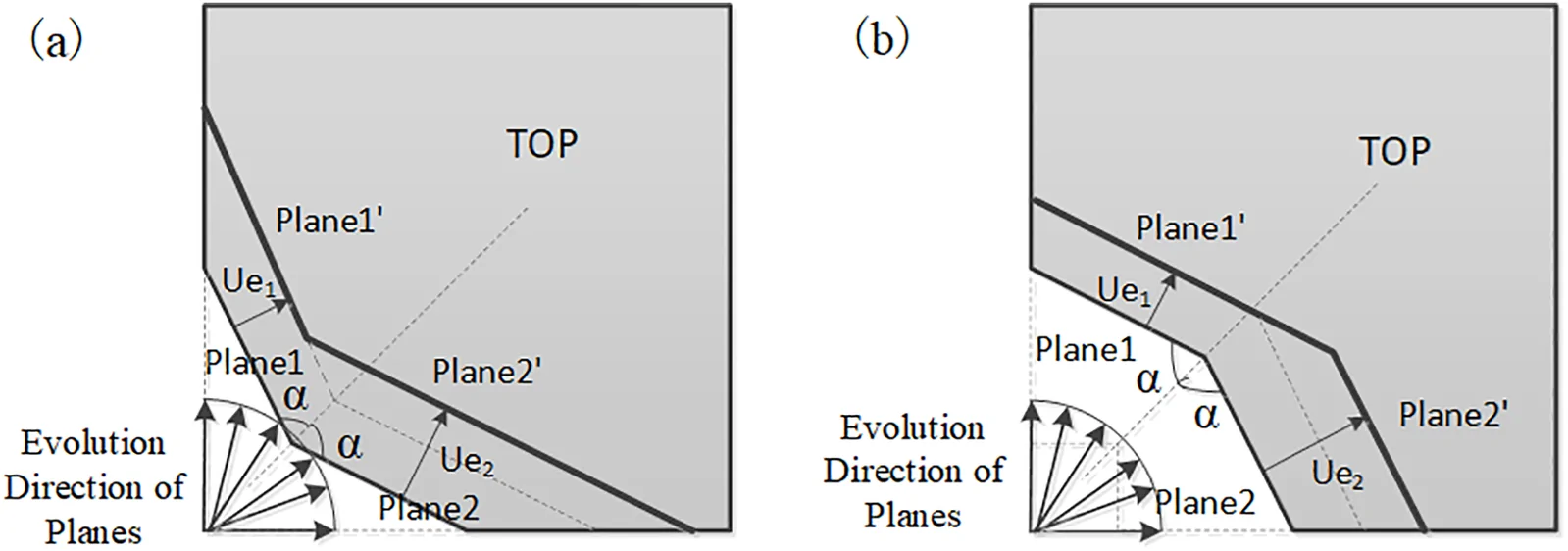
Evolution process of crystal planes at the intersection of mask boundaries: (a) Convex corner; (b) Concave corner.

For the upper contour evolution, there are mainly two types: convex corner etching and concave corner etching. Among them, the convex-etched profiles are predominantly formed by the planes with the maximum undercutting rate perpendicular to the mask, while the concave-etched profiles are the opposite. As shown in Fig 3(in the uploaded file), when Ue_1_ = Ue_2_, Plane1 and Plane2 evolve synchronously to form a structural plane. When Ue_1_ < Ue_2_, Plane2 dominates at the convex corner, and Plane1 gradually disappears during the etching process; at the concave corner, Plane1 dominates, while Plane2 is excluded and disappears.

In order to clarify the etching process and rules of above microstructure, this study selected the 71℃ 40wt% KOH solution (stirring) as the etching condition and Si (100) mask chip as the etching object. Among them, the mask shapes are window and convex masks with boundaries parallel to the < 110 > . After 60 minutes of etching, the following etching results were obtained.

1) Analysis of the etching process of window mask

**Fig 4:**
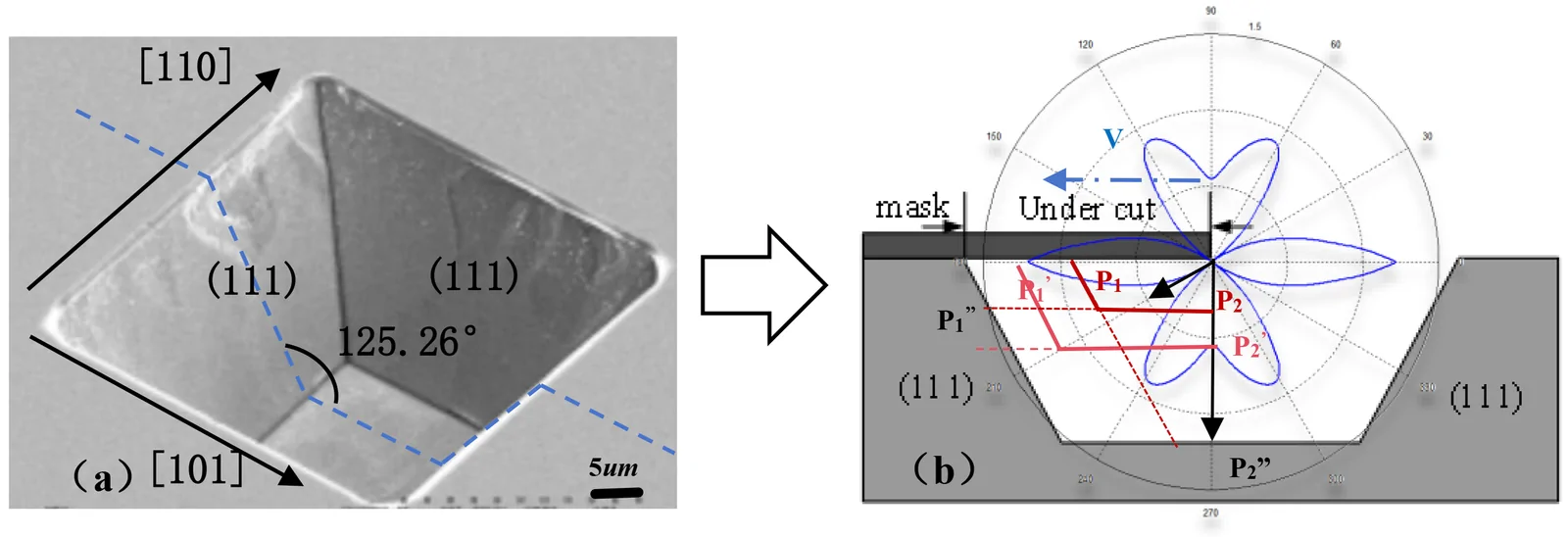
The sidewall etching process of the window mask:(a) shape and etching results of the window mask after 60 minutes etching; (b) etching results of the sidewall structure at different stages.

As shown in Fig 4 (a)(in the uploaded file), the etching structure of the Si (100) window mask is a truncated pyramid cavity, with an angle of 125.26°between the isosceles plane and the bottom plane, and all structural planes are relatively smooth and flat. According to the angle relationship between crystal planes and combined with the etching rate curve in Fig 1 (c), it can be seen that all four isosceles structural planes are Si(111) and the bottom plane is Si(100). In order to explore the etching process of the mask sidewall, the following works were carried out in this study: firstly, the etching rates of all crystal planes parallel to <110 > direction in Fig 1 (a) were extracted and transformed into polar coordinates to obtain Fig 4 (b)(in the uploaded file).

According to the aforementioned cross-sectional polygon evolution theory, this study marks the crystal orientation of the plane with the minimum etching rate using arrows and make the mask boundary as the curvature center of the polar coordinate rate. Finally, the etched profiles will be formed by the competition between various planes that are locally extremely close to the center of curvature. It can be seen from Fig 4 (b), the crystal planes with extremely low rates are P1 and P2 which form the initial profiles in the early stage of etching. However, the etching rate of the P2 is much higher than that of P1. Due to the advantage of etching rate, the P1will displace the faster P2 during subsequent etching, resulting in the formation of a truncated pyramid cavity.

2) Analysis of the etching process of mesa mask

**Fig 5:**
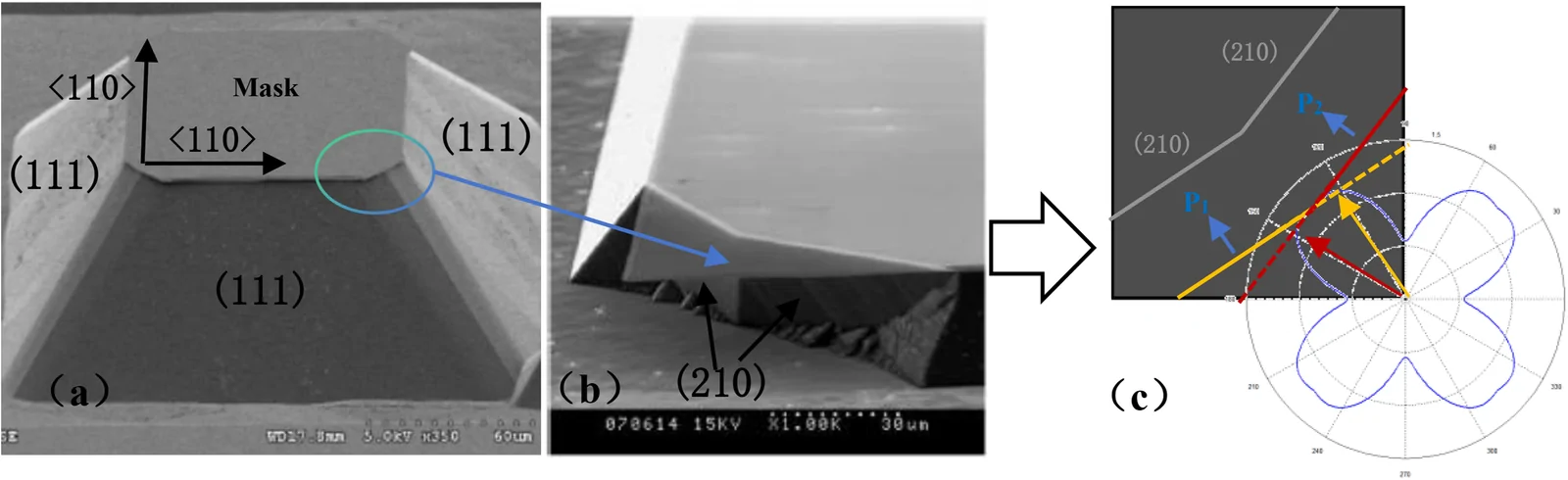
The etching process of convex corners in mesa mask: (a) Shape and etching results of mesa mask after 60 minutes etching; (b) Initial convex corner etching results after 15 minutes etching; (c) Dynamic adjustment process of convex mask boundary.

As shown in Fig 5 (a)(in the uploaded file), the etching structure of Si (100) mesa mask is a truncated pyramids, and each profile is the smooth Si(111). As well as, there are regular polygonal surfaces at the convex corners of the mask. The etching process of the mesa mask is determined by the etching of two parts, namely: 1. Etching at the mask boundary; 2. Etching at the convex corners. Among them, the etching at the mask boundary occurs in the middle of the two convex corners, and the etching process is consistent with the cross-sectional polygon evolution; the etching process at the convex corner is relatively complex, involving two stages:the upper contour evolution and the cross-sectional polygon evolution.The initial etching of the convex corner vertex creates new mask boundaries, which progressively migrate inward during the process. This profile formation aligns with the upper contour evolution process. Meanwhile, mask boundaries formed at any stage of the upper contour evolution undergo etching according to the cross-sectional polygon evolution process. These two interdependent etching processes continuously modify the sidewall until the etching completes. As shown in Fig 5 (b)(in the uploaded file), the originally two < 110 > mask boundaries perpendicular to each other in the early etching stage are pushed inward to form obtuse edges and (210) sidewalls. The dynamic change process of the mask boundary at the convex corner is shown in Fig 5 (c)(in the uploaded file), which means that after converting the polar coordinates of the etching rates of all crystal planes perpendicular to the (100) crystal plane, the crystal plane with the maximum etching rate, namely (210), dominates to form the etching sidewall and continuously advances forward.

## 3. The 3D Etching Simulation Model Based on Level Set Method

**Fig 6:**
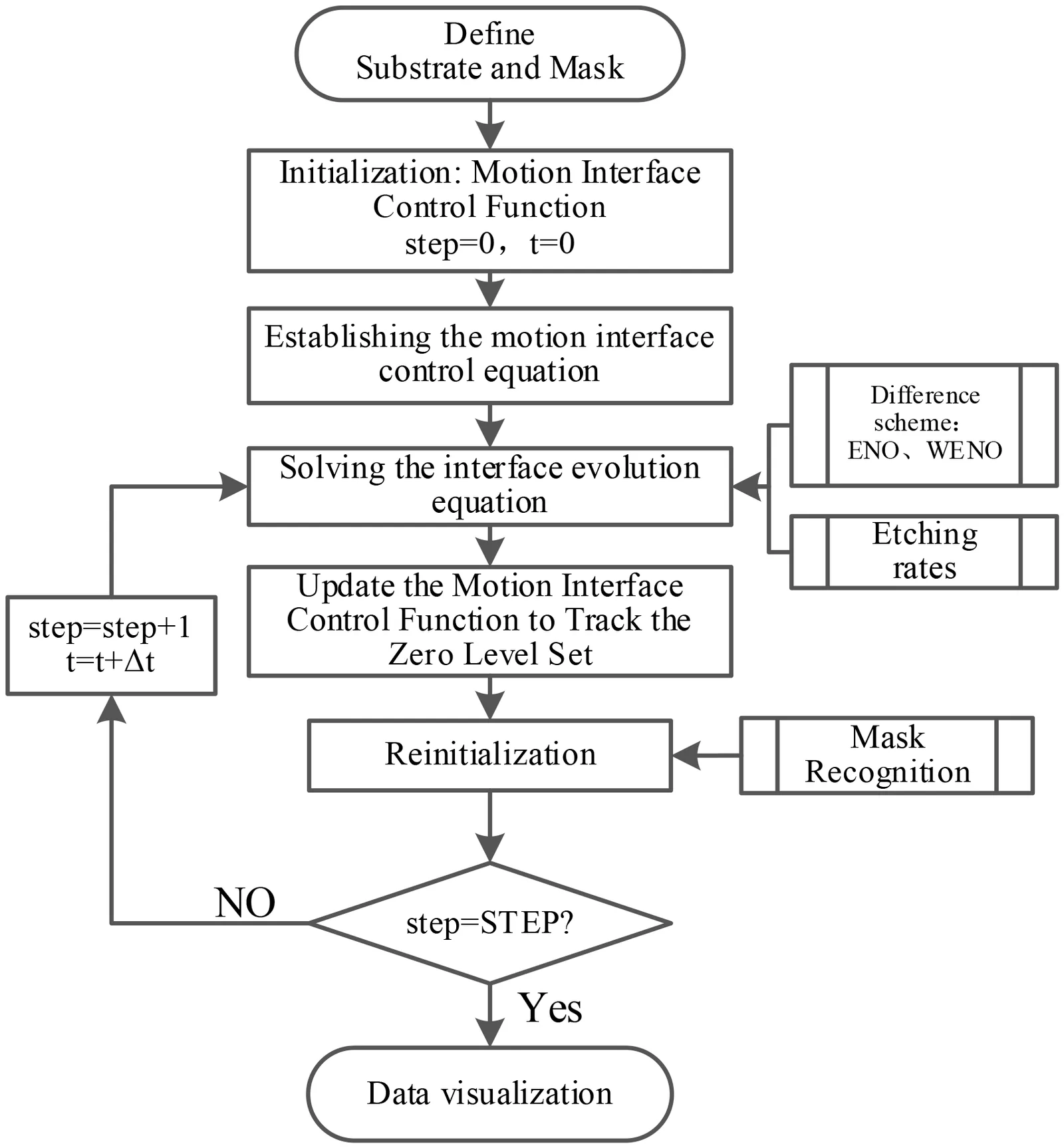
The algorithm flowchart of the etching profile simulation based on the Level-Set method.

Based on the anisotropic wet etching shape evolution rule, the etching interface of crystal silicon is regarded as the zero contour of Level Set function at a certain time, and the evolution process of etching surface is transformed into the implicit function equation of Level Set function in four-dimensional space [3, 17, 20, 26, 29]. In developing the Level Set etching model, this study imposed the following constraints: 1) During motion interface tracking, the symbol distance function must be satisfied in the vicinity of the zero contour. 2) During initialization, the symbol distance function is first satisfied near the zero contour when the number of iterations is small. 3) The mask is treated as part of the silicon and consistently maintained as the zero contour. Fig 6 (in the uploaded file) shows the algorithm flowchart of the anisotropic etching profile simulation for silicon based on the Level-Set method, with the numerical simulation program developed using MATLAB software.

### 3.1. Level-Set Equation

This study defines a Level set function φ(x,t), where the set of points satisfying φ(x,t)=0 defines the interfaceΓ(t), and represents the position vector in n-dimensional space. When tracking etching motion interfaces, the Level set function is continuously updated in space according to a predefined rule and the position of its zero-level set is determined，and the resulting surface obtained in this way corresponds to the evolved shape.The Level set functionφ(x,t) is defined as:


$$\begin{array}{c} \phi (x,t=\text{0})=\left\{ \begin{array}{c} @ld(x),\;x\in \Omega^{+}\\ \text{0},\;x\in \partial \Omega =\Gamma (\text{0})\\ -d(x),\;x\in \Omega^{-} \end{array}\right. \end{array}$$ (3)

The above equation represents the problem of the zero isosurfaceΓ(t) related to the spatial variable x at a certain time t, i.e., {x|φ(x,t) = 0}. Among them, when the function φ(x,t)=0, x is located on the interface Γ(t)； φ(x,t)=d(x)>0, x is located on the outer side of interface Γ(t); φ(x,t)=−d(x)<0, x is located on the inner side of interfaceΓ(t); namely, φ(x,t) is the symbol distance function relative to the motion interface. The Level Set functionφ(x,t)’s contour lines evolve over time driven by velocity fields, with common types including the normal velocity fieldVN, external vector field $\vec{\text{S}}$, and curvature-dependent velocity field u. The general partial differential equation governing Level Set function evolution is expressed in Equation 3.2 [30, 31].


$$\begin{array}{c} \frac{\partial \varphi }{\partial t}+\vec{S}⬝\nabla \varphi +V_{N}\left|\nabla \varphi \right|=u\left|\nabla \varphi \right| \end{array}$$ (4)

Here, ∇φdenotes the function gradient, and|∇φ|represents the gradient magnitude. Since the velocity field of the crystal etching only exists in the normal direction, the simplified motion interface control equation can be obtained:


$$\begin{array}{c} \frac{\partial \varphi }{\partial t}+V_{N}\left|\nabla \varphi \right|=0 \end{array}$$ (5)

Based on the initial zero equivalent surface{x|φ(x,t) = 0}, the tangent plane will be moved forward along the normal direction by a certain etching distance（L=VN*t）, and the zero equivalent surface obtained at this time is the etching envelope surface.The φ(x,t) function requires sufficient smoothness and must exhibit monotonic normality near the zero-value surface. Thus, it can be expressed as a symbol distance function relative to the moving interface, satisfying the condition |∇φ|=1. Consequently, Equation (3.3) can be simplified to:


$$\begin{array}{c} \frac{\partial \varphi }{\partial t}=-V_{N} \end{array}$$ (6)

### 3.2. The interface evolution equation

#### 3.2.1. Difference scheme.

Equation 3.3 is a time-dependent first-order hyperbolic partial differential equation, representing a specific form of the Hamilton-Jacobi equation $\varphi_{t}+H(\nabla \varphi )=0$[17]. The Hamiltonian function is defined by the boundary condition $H(\nabla \varphi )=V_{n}|\nabla \varphi |$, enabling numerical solution via finite difference methods.This study employed the finite difference method using the Lax-Friedrichs (LF) scheme [30, 31] for numerical solution. The LF scheme can be expressed as Equation (3.5).


$$\begin{array}{c} \varphi_{ijk}^{n+1}=\varphi_{ijk}^{n}-\Delta t\left[H\left(\frac{\prod \text{X}}{\text{2}},\frac{\prod \text{Y}}{\text{2}},\frac{\prod \text{Z}}{\text{2}})-\frac{\text{1}}{\text{2}}(\alpha_{x}(\prod \text{X})+\alpha_{y}(\prod \text{Y})+\alpha_{z}(\prod \text{Z})\right)\right] \end{array}$$ (7)

In the above formula:$\prod \text{X}=\varphi_{ijk}^{+x}-\varphi_{ijk}^{-x}$, $\prod \text{Y}=\varphi_{ijk}^{+\text{y}}-\varphi_{ijk}^{-y}$, $\prod \text{Z}=\varphi_{ijk}^{+z}-\varphi_{ijk}^{-z}$。

Among，$\alpha_{x},\alpha_{y},\alpha_{z}$and as follow:


$$\begin{array}{c} \left\{ \begin{array}{c} @l@H(\nabla \varphi )=V_{N}=V_{N}\sqrt{\varphi_{x}^{\text{2}}+\varphi_{y}^{\text{2}}+\varphi_{z}^{\text{2}}}\\ \alpha_{x}=max\left|\frac{\partial H}{\partial \varphi_{x}}\right|,\alpha_{y}=max\left|\frac{\partial H}{\partial \varphi_{y}}\right|,\alpha_{z}=max|\frac{\partial H}{\partial \varphi_{z}} \end{array}\right.| \end{array}$$ (8)

Assuming no correlation between$V_{N}$and$\varphi_{x}$,$\varphi_{y}$,$\varphi_{z}$， the parameter$\alpha_{x}$,$\alpha_{y}$,$\alpha_{z}$can be simplified to Equation (3.7).


$$\begin{array}{c} \alpha_{x}=max\left|u\right|,\alpha_{y}=max\left|v\right|,\alpha_{z}=max\left|w\right| \end{array}$$ (9)

In this formula, u, v, and w represent the x, y, and z components of the normal velocity on the crystal plane. As the dependent region of the difference scheme in this study overlaps with the initial value problem’s region for the partial differential equation, the CFL (Courant-Friedrichs-Lewy) condition is applied to restrict the time step Δt, thereby enhancing the it’s calculation power. Specifically:


$$\begin{array}{c} \Delta t⬝max(\alpha_{x}+\alpha_{y}+\alpha_{z})<\alpha \end{array}$$ (10)

In the above equation, α∈(0,1). Equations (3.5) to (3.8) can be used to achieve a stable numerical solution for equation (3.4). During the simulation, the φ(x,t) function is continuously updated over time to track the movement of the interface during wet etching of silicon, ultimately enabling the simulation of etching profile.

#### 3.2.2.Spatial discretization scheme.

1）ENO Scheme

To ensure sufficient computational accuracy and achieve sufficiently sharp edges in the simulated graphics, this study employs the ENO (Essentially Non-Oscillatory) and WENO (Weighted-Essentially Non-Oscillatory) as high-precision difference schemes in the spatial direction [32], while the Euler forward difference scheme is used in the temporal direction. The subset of{xi−3,xi−2, xi−1,xi,xi+1,xi+2,xi+3} is selected as the template to construct the 3-order ENO scheme for $(\varphi_{x}^{+}{)}_{i}$$(\varphi_{x}^{\text{-}}{)}_{i}$, as shown in equations (3.9) and (3.10).


$$\begin{array}{c} (\varphi_{x}^{-}{)}_{i}^{\text{3}}=\left\{ \begin{array}{c} @l@\frac{\text{1}}{\text{3}}\frac{\varphi_{i-2}-\varphi_{i-3}}{\Delta x}-\frac{\text{7}}{\text{6}}\frac{\varphi_{i-1}-\varphi_{i-2}}{\Delta x}+\frac{11}{\text{6}}\frac{\varphi_{i}-\varphi_{i-1}}{\Delta x},\text{model:}\{x_{i-3},x_{i-2},x_{i-1},x_{i}\}\\ -\frac{\text{1}}{\text{6}}\frac{\varphi_{i-1}-\varphi_{i-2}}{\Delta x}+\frac{\text{5}}{\text{6}}\frac{\varphi_{i}-\varphi_{i-1}}{\Delta x}+\frac{\text{1}}{\text{3}}\frac{\varphi_{i+1}-\varphi_{i}}{\Delta x},\text{model:}\{x_{i-2},x_{i-1},x_{i},x_{i+1}\}\\ \frac{\text{1}}{\text{3}}\frac{\varphi_{i}-\varphi_{i-1}}{\Delta x}+\frac{\text{5}}{\text{6}}\frac{\varphi_{i+1}-\varphi_{i}}{\Delta x}-\frac{\text{1}}{\text{6}}\frac{\varphi_{i+2}-\varphi_{i+1}}{\Delta x},\text{model:}\{x_{i-1},x_{i},x_{i+1},x_{i+2}\} \end{array}\right. \end{array}$$ (11)

$$\begin{array}{c} (\varphi_{x}^{+}{)}_{i}^{\text{3}}=\left\{ \begin{array}{c} @l@\frac{\text{1}}{\text{3}}\frac{\varphi_{i+3}-\varphi_{i+2}}{\Delta x}-\frac{\text{7}}{\text{6}}\frac{\varphi_{i+2}-\varphi_{i+1}}{\Delta x}+\frac{11}{\text{6}}\frac{\varphi_{i+1}-\varphi_{i}}{\Delta x},\text{model:}\{x_{i},x_{i+1},x_{i+2},x_{i+3}\}\\ -\frac{\text{1}}{\text{6}}\frac{\varphi_{i+2}-\varphi_{i+1}}{\Delta x}+\frac{\text{5}}{\text{6}}\frac{\varphi_{i+1}-\varphi_{i}}{\Delta x}+\frac{\text{1}}{\text{3}}\frac{\varphi_{i}-\varphi_{i-1}}{\Delta x},\text{model:}\{x_{i-1},x_{i},x_{i+1},x_{i+2}\}\\ \frac{\text{1}}{\text{3}}\frac{\varphi_{i+1}-\varphi_{i}}{\Delta x}+\frac{\text{5}}{\text{6}}\frac{\varphi_{i}-\varphi_{i-1}}{\Delta x}-\frac{\text{1}}{\text{6}}\frac{\varphi_{i-1}-\varphi_{i-2}}{\Delta x},\text{model:}\{x_{i-2},x_{i-1},x_{i},x_{i+1}\} \end{array}\right. \end{array}$$ (12)

2) WENO Scheme

The WENO scheme is constructed by weighting the difference of the 3-order ENO formula, and the contribution of each interpolation to the WENO interpolation is distinguished by the weight [30], so as to reduce the error and improve the accuracy.

Construct$(\varphi_{x}^{-}{)}_{i}$ WENO format, define:


$$\begin{array}{c} v_{\text{1}}=D^{-}\varphi_{i-2},v_{\text{2}}=D^{-}\varphi_{i-1},v_{\text{3}}=D^{-}\varphi_{i},v_{\text{4}}=D^{-}\varphi_{i+1},v_{\text{5}}=D^{-}\varphi_{i+2} \end{array}$$ (13)

In the above formula:$D^{-}\varphi_{i}=(\varphi_{i}-\varphi_{i-1})/\Delta x$。

Substituting Equation (11) into Equation (9) yields three 3-order ENO scheme approximations for $(\varphi_{x}^{-}{)}_{i}$:


$$\begin{array}{c} \left\{ \begin{array}{c} \phi_{x}^{\text{1}}=\frac{\nu_{\text{1}}}{\text{3}}-\frac{\text{7}\nu_{\text{2}}}{\text{6}}+\frac{\text{11}\nu_{\text{3}}}{\text{6}}\\ \phi_{x}^{\text{2}}=-\frac{\nu_{\text{2}}}{\text{6}}+\frac{\text{5}\nu_{\text{3}}}{\text{6}}+\frac{\nu_{\text{4}}}{\text{3}}\\ \;\;\;\;\;\;\phi_{x}^{\text{3}}=\frac{\nu_{\text{5}}}{\text{3}}+\frac{\text{5}\nu_{\text{4}}}{\text{6}}-\frac{\nu_{\text{5}}}{\text{6}} \end{array}\right. \end{array}$$ (14)

The WENO scheme approximation formula for $(\varphi_{x}^{-}{)}_{i}$is a convex combination of three approximate formulas in equation (3.12), where ${\text{0}<\omega }_{\text{k}}<\text{1}$ (k = 1，2，3) and satisfies $\omega_{\text{1}}+\omega_{\text{2}}+\omega_{\text{3}}=\text{1}$。


$$\begin{array}{c} \phi_{\text{x}}=\omega_{\text{1}}\phi_{\text{x}}^{\text{1}}+\omega_{\text{2}}\phi_{\text{x}}^{\text{2}}+\omega_{\text{3}}\phi_{\text{x}}^{\text{3}} \end{array}$$ (15)

To determine the weights $\omega_{\text{k}}$, the smoothness of the three approximate formula templates in Equation (12) is evaluated separately.


$$\begin{array}{c} \left\{ \begin{array}{c} @l@S_{\text{1}}=\frac{13}{12}(v_{\text{1}}-2v_{\text{2}}+v_{\text{3}}{)}^{\text{2}}+\frac{\text{1}}{\text{4}}(v_{\text{1}}-4v_{\text{2}}+3v_{\text{3}}{)}^{\text{2}}\\ S_{\text{2}}=\frac{13}{12}(v_{\text{2}}-2v_{\text{3}}+v_{\text{4}}{)}^{\text{2}}+\frac{\text{1}}{\text{4}}(v_{\text{2}}-v_{\text{4}}{)}^{\text{2}}\\ S_{\text{3}}=\frac{13}{12}(v_{\text{3}}-2v_{\text{4}}+v_{\text{5}}{)}^{\text{2}}+\frac{\text{1}}{\text{4}}(3v_{\text{3}}-4v_{\text{4}}+v_{\text{5}}{)}^{\text{2}} \end{array}\right. \end{array}$$ (16)

While evaluating the smoothness of the template, the parameter expressions (3.14) and (3.15) [30] are defined. Based on these two expressions, the calculation formulas (3.16) for each weight can be obtained, ultimately yielding the approximate value of the WENO scheme for $(\varphi_{x}^{-}{)}_{i}$.


$$\begin{array}{c} \alpha \text{1}=\frac{0.1}{\left(S_{\text{1}}+\varepsilon \right)},\alpha \text{2}=\frac{0.6}{\left(S_{\text{2}}+\varepsilon \right)},\alpha \text{3}=\frac{0.3}{\left(S_{\text{1}}+\varepsilon \right)} \end{array}$$ (17)

$$\begin{array}{c} \varepsilon =10^{-6}max\{v_{\text{1}}^{\text{2}},v_{\text{2}}^{\text{2}},v_{\text{3}}^{\text{2}},v_{\text{4}}^{\text{2}},v_{\text{5}}^{\text{2}}\}+10^{-99} \end{array}$$ (18)

$$\begin{array}{c} w_{\text{1}}=\frac{\alpha_{\text{1}}}{\alpha_{\text{1}}+\alpha_{\text{2}}+\alpha_{\text{3}}},w_{\text{2}}=\frac{\alpha_{\text{2}}}{\alpha_{\text{1}}+\alpha_{\text{2}}+\alpha_{\text{3}}},w_{\text{3}}=\frac{\alpha_{\text{3}}}{\alpha_{\text{1}}+\alpha_{\text{2}}+\alpha_{\text{3}}} \end{array}$$ (19)

The WENO approximation scheme for $(\varphi_{x}^{+}{)}_{i}$ is constructed by following the procedure of WENO scheme for $(\varphi_{x}^{-}{)}_{i}$, with the following definitions.


$$\begin{array}{c} v_{\text{1}}=D^{+}\varphi_{i+2},v_{\text{2}}=D^{+}\varphi_{i+1},v_{\text{3}}=D^{+}\varphi_{i},v_{\text{4}}=D^{+}\varphi_{i-1},v_{\text{5}}=D^{+}\varphi_{i-2} \end{array}$$ (20)

In the above formula:$D^{+}\varphi_{i}=(\varphi_{i+1}-\varphi_{i})/\Delta x$。

Based on the three 3-order ENO approximations of$(\varphi_{x}^{+}{)}_{i}$ in Equation (3.10) and equations (3.14) to (3.17), the WENO approximation of $(\varphi_{x}^{+}{)}_{i}$ can be expressed as the form shown in Equation (3.12).

#### 3.2.3.Reinitialization.

To ensure solution accuracy, the φ(x,t) function must remain a symbol distance function, requiring initialization at each time step. Two conditions govern the Level Set function’s initialization: (1) Zero contour positions must remain unchanged; (2) The function must satisfy the symbol distance property [38]. Accordingly, this study employs a partial differential equation-based implicit iteration method to formulate the following Hamilton-Jacobi-type partial differential equation:


$$\begin{array}{c} \left\{ \begin{array}{c} @l@\partial d/\partial t+s\left(d_{\text{0}}\right)(\left|\nabla d\right|-1)=0\\ s\left(d_{\text{0}}\right)=\frac{d_{\text{0}}}{\sqrt{{d_{\text{0}}}^{\text{2}}+(\left|\nabla d\right|\Delta x{)}^{\text{2}}}}\\ d\left(x,0\right)=d_{\text{0}}\left(x\right)=\varphi \left(x,t\right) \end{array}\right. \end{array}$$ (21)

In the above formula: d=d(x,t), $x\in {\text{R}}^{\text{n}}$, t∈[0,T]。

When |∇d|=1, ∂d/∂t=0, and the equation approaches a stable solution. Setting φ=d, the Level Set function becomes a signed distance function. Moreover, the moving interface (zero contour) satisfies dφ/dt=0, ensuring the position of the zero contour remains unchanged during reinitialization. For the numerical solution of the reinitialization equation, the modified Godunov scheme is used to perform the initialization.

Equation 3.18 is rewritten as follows:


$$\begin{array}{c} d_{\tau }+\left(\frac{S\left(d_{\text{0}}\right)d_{x}}{\sqrt{d_{x}^{\text{2}}+d_{y}^{\text{2}}+d_{z}^{\text{2}}}}\right)d_{x}+\left(\frac{S\left(d_{\text{0}}\right)d_{y}}{\sqrt{d_{x}^{\text{2}}+d_{y}^{\text{2}}+d_{z}^{\text{2}}}}\right)d_{y}+\left(\frac{S\left(d_{\text{0}}\right)d_{z}}{\sqrt{d_{x}^{\text{2}}+d_{y}^{\text{2}}+d_{z}^{\text{2}}}}\right)d_{z}=S\left(d_{\text{0}}\right) \end{array}$$ (22)

Define:


$$\begin{array}{c} S=\frac{S\left(d_{\text{0}}\right)(\left|d_{m}^{+}\right|-\left|d_{m}^{-}\right|)}{d_{m}^{+}-d_{m}^{-}} \end{array}$$ (23)

Discrete the following for$d_{m}$ (m = x,y,z):


$$\begin{array}{c} d_{m}=\left\{ \begin{array}{c} @l@d_{x}^{-}\;S\left(d_{\text{0}}\right)d_{m}^{+}\geq 0,S\left(d_{\text{0}}\right)d_{m}^{-}\geq 0\\ d_{m}^{+}\;S\left(d_{\text{0}}\right)d_{m}^{+}\leq 0,S\left(d_{\text{0}}\right)d_{m}^{-}\leq 0\\ 0\;S\left(d_{\text{0}}\right)d_{m}^{+}>0,S\left(d_{\text{0}}\right)d_{m}^{-}<0\\ d_{m}^{-}\;S\left(d_{\text{0}}\right)d_{m}^{+}<0,S\left(d_{\text{0}}\right)d_{m}^{-}>0,S>0\\ d_{m}^{+}\;S\left(d_{\text{0}}\right)d_{m}^{+}<0,S\left(d_{\text{0}}\right)d_{m}^{-}>0,S\leq 0 \end{array}\right. \end{array}$$ (24)

To improve accuracy, the discrete values of the left and right derivatives constructed by the WENO scheme are taken as $d_{m}^{-},d_{m}^{+}$. The time derivative is discretized using a first-order Euler forward difference quotient.

### 3.3. Space Coordinate System Transformation

Since the coordinate system of the etching simulation system is based on the substrate system, the crystal plane evolution rate must correspond to the crystal orientation. When the wafer’s tangent is not perpendicular to the Z-axis, a coordinate system transformation is required to convert the gradient calculation results from the substrate system to the crystal system. In this paper, the substrate system is denoted as x’ y ‘z,’ and the crystal system as x y z. The substrate system gradient is$\Delta =a\text{'}\vec{i}\text{'}+b\text{'}\vec{j}\text{'}+c\text{'}\vec{k}\text{'}$ with the gradient vector (a ‘, b,’ c’), while the crystal system gradient is$\Delta =a\vec{i}+b\vec{j}+c\vec{k}$, with the gradient vector changed to (a, b, c).

**Fig 7:**
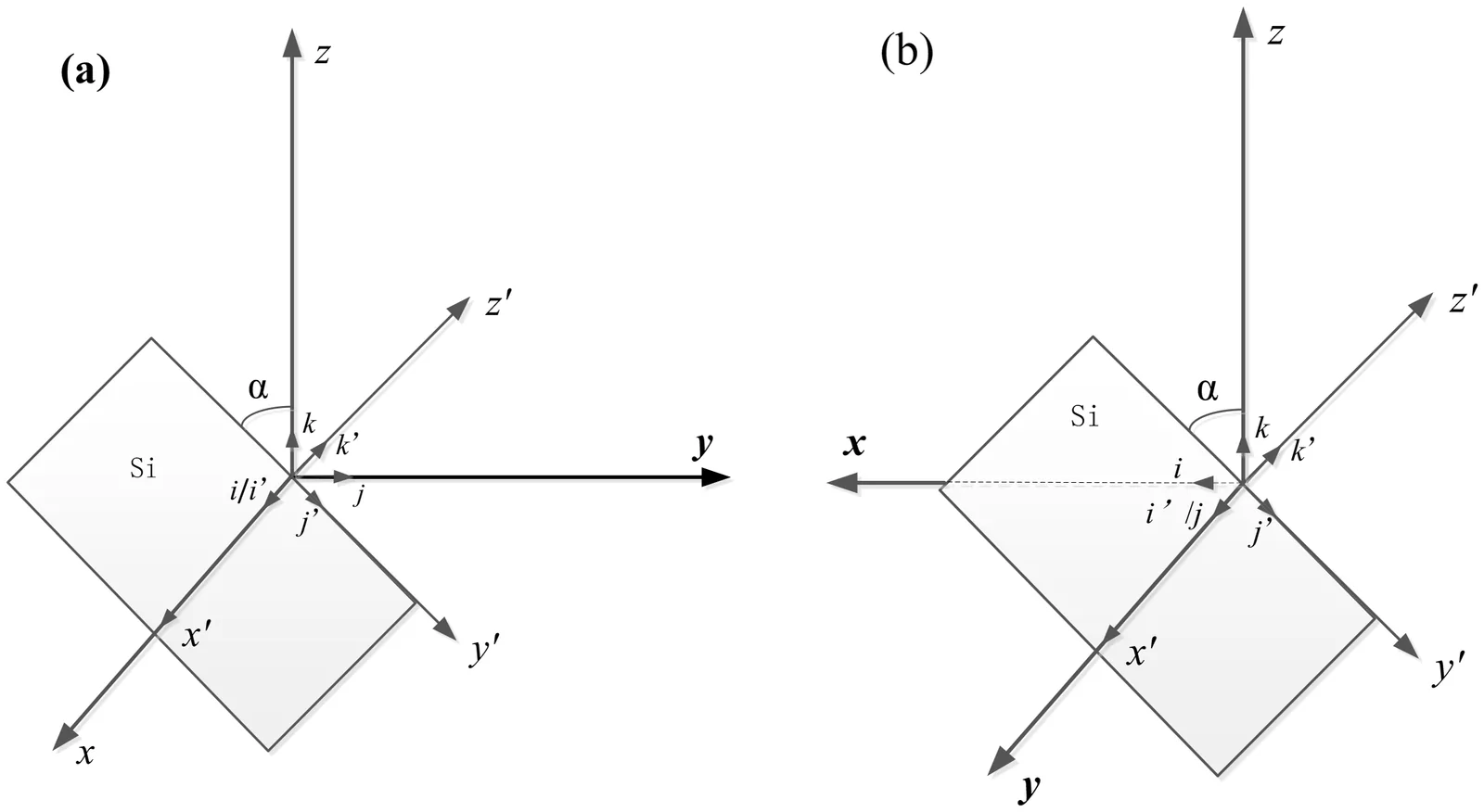
Schematic diagram of chip tangential rotation around coordinate axes: (a) rotation around the x-axis, (b) rotation around the y-axis.


If the chip rotates tangentially around the x-axis by an angle α, as shown in Fig 7(a)(in the uploaded file), the coordinate transformation of the gradient vector is as follows:

$$\begin{array}{c} \left\{ \begin{array}{c} @l@a=a\text{'}\\ b=b\text{'}sin\alpha +c\text{'}cos\alpha \\ c=c\text{'}sin\alpha -b\text{'}cos\alpha \end{array}\right. \end{array}$$ (25)

If the chip rotates tangentially around the y-axis by an angle α, as shown in Fig 7(b)(in the uploaded file), the coordinate transformation of the gradient vector is as follows:


$$\begin{array}{c} \left\{ \begin{array}{c} @l@a=a\text{'}\\ b=b\text{'}sin\alpha -c\text{'}cos\alpha \\ c=c\text{'}sin\alpha +b\text{'}cos\alpha \end{array}\right. \end{array}$$ (26)

### 3.4. Speed modeling and 3D etching profile simulation

**Fig 8:**
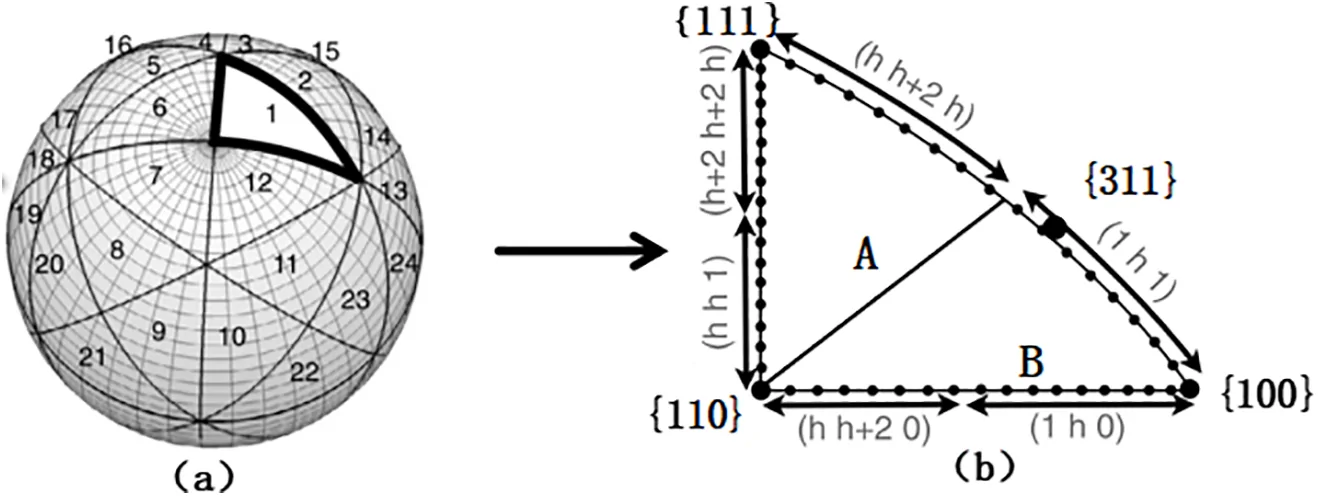
(a) Equivalent regions on the unit sphere according to the crystallographic symmetry of silicon; (b)Correspondence between the crystallographic orientations along the boundary of region 1 and well-known surface families [26].

To reconstruct the complete etching rate distribution, the silicon hemisphere is divided into 24 regions, each corresponding to a curved triangular zone delimited by three neighboring representatives of the main crystallographic orientations {110}, {100}, and {111}. The etching rate of the hemisphere is then obtained through interpolation based on the etching rates of these three crystal planes [26]. However, as shown in Fig 1(c), the etching rate curve of the entire single-crystal silicon surface also exhibits a maximum etching rate at the {311} crystal plane. To further improve the accuracy of the interpolation, this study subdivides the triangular regions enclosed by {110}, {100}, and {111} into two areas: Area A (containing {110}, {100}, and {311}) and Area B (containing {311}, {110}, and {111}), as illustrated in Fig 8(in the uploaded file).

In order to obtain the whole etching rate of silicon planes, the velocity vector of the key crystal planes in the above two regions are taken as unit vector, and the original orthogonal coordinate system is replaced by a new non-orthogonal coordinate system with non-orthogonal unit vector, and then the 3D vector interpolation calculation is carried out to realize the etching rate of a few typical crystal surface to simulate the etching rate of any crystal surface in space.

1) Coordinate transformation

Using the velocity vectors of the four typical crystal planes mentioned above, a new non orthogonal coordinate system (a, b, c) is constructed through coordinate transformation, with the following formula:


$$\begin{array}{c} \left[\begin{array}{ccc} @ccc@a & b & c \end{array}\right]=\left[\begin{array}{ccc} @ccc@x & y & z \end{array}\right]*{\left[\begin{array}{ccc} @ccc@h_{\text{1}} & k_{\text{1}} & l_{\text{1}}\\ h_{\text{2}} & k_{\text{2}} & l_{\text{2}}\\ h_{\text{3}} & k_{\text{3}} & l_{\text{3}} \end{array}\right]}^{-1}\left[\begin{array}{ccc} @ccc@s_{\text{1}} & 0 & 0\\ 0 & s_{\text{2}} & 0\\ 0 & 0 & s_{\text{3}} \end{array}\right] \end{array}$$ (27)

Among them, [a, b, c] are the coordinate values of the new coordinate system, [x, y, z] are the coordinate values of the original coordinate system, and the transformation matrix ${\left[\begin{array}{ccc} h_{\text{1}} & k_{\text{1}} & l_{\text{1}}\\ h_{\text{2}} & k_{\text{2}} & l_{\text{2}}\\ h_{\text{3}} & k_{\text{3}} & l_{\text{3}} \end{array}\right]}^{-1}$ is an inverse matrix composed of key vectors determined by the Miller index (hkl) of three typical crystal planes. The scaling matrix $\left[\begin{array}{ccc} s_{\text{1}} & 0 & 0\\ 0 & s_{\text{2}} & 0\\ 0 & 0 & s_{\text{3}} \end{array}\right]$ is a diagonal matrix composed of the maximum values of typical crystal plane vectors in a coordinate system. Its function is to scale up or down existing vectors in order to convert them into unit vectors. The two non-orthogonal three-dimensional coordinate system transformations are as shown in Table 1:

**Table 1:** Transformation Relationships of Coordinate Systems in Two Non Orthogonal Three Dimensional Coordinate Regions.

transformation matrix	scaling matrix	coordinate transformation	normalization factor
$\left[\begin{array}{ccc} @ccc@1 & 0 & 0\\ 1 & 1 & 0\\ 3 & 1 & 1 \end{array}\right]$	$\left[\begin{array}{ccc} @ccc@1 & 0 & 0\\ 0 & 1 & 0\\ 0 & 0 & 3 \end{array}\right]$	*a=x-y-2z* *b=y-z* *c=3z*	*1/x*
$\left[\begin{array}{ccc} @ccc@1 & 1 & 1\\ 1 & 1 & 0\\ 3 & 1 & 1 \end{array}\right]$	$\left[\begin{array}{ccc} @ccc@1 & 0 & 0\\ 0 & 1 & 0\\ 0 & 0 & 3 \end{array}\right]$	*a=(y-x)/2+z* *b=y-z* c=3/2*(x-y)	*1/x*

2) The plane etching rate obtained by interpolating calculation

Through the above transformation, any vector in the (x, y, z) coordinate system can be transformed into the sum of velocity vectors on three key crystal planes and represented as the unit vector coordinate. When the components in the above three directions are multiplied by the rates of these three typical crystal plane directions, the etching rate of the crystal plane at any position in the three-dimensional coordinate system can be obtained. In order to reduce computational workload, this study takes the etching rate of the Si(111) as the standard rate, and divides the simulated crystal plane rate in any direction by it to obtain the relative rateR(x,y,z), that is [8]:


$$\begin{array}{c} R\left(x,y,z\right)=N*\left(a*W_{\text{1}}+b*W_{\text{2}}+c*W_{\text{3}}\right)*R_{111} \end{array}$$ (28)

Among them, N is the normalization factor, R111 is the etching rate of the (111), and W1, W2, and W3 are the ratio of the etching rate of the three typical crystal planes that make up the non orthogonal three-dimensional coordinate region to the R111 rate. The formula for calculating the interpolation rate of crystal planes within two non-orthogonal three-dimensional coordinate regions is obtained, as shown in the following Table 2:

**Table 2:** Interpolation calculation formula for etching rate in six non orthogonal three-dimensional coordinate regions.

Unit vector	Sphere range	Calculating formula for the interpolant rate
[100] [110] [311]	0≤θ≤arctan(1/3) 0≤φ≤arctan(sinθ)	$R\left(x,y,z\right)=(1/x)*\left[(x-y-2z)*R_{100}+(y-z)*R_{110}+3z*R_{311}\right]$ $R\left(\theta ,\varphi \right)=\left(1-tan\theta -2*\frac{tan\varphi }{cos\theta }\right)*R_{100}+\left(tan\theta -\frac{tan\varphi }{cos\theta }\right)*R_{110}+3*\frac{tan\varphi }{cos\theta }*R_{311}$
	arctan(1/3)≤θ≤(π/4) 0≤φ≤arctan(cosθ−sinθ)/2	
[111] [110] [311]	arctan(1/3)≤θ≤(π/4) φ≥arctan(cosθ−sinθ)/2 φ≤arctan(sinθ)	$R\left(x,y,z\right)=\frac{\text{1}}{x}*\left\{[\frac{\text{1}}{\text{2}}*(y-x)+z]*R_{111}+(y-z)*R_{110}+\frac{\text{3}}{\text{2}}*(x-y)*R_{311}\right\}$ $R\left(\theta ,\varphi \right)=\left(\frac{\text{1}}{\text{2}}*tan\theta +\frac{tan\varphi }{cos\theta }-\frac{\text{1}}{\text{2}}\right)*R_{111}+\left(tan\theta -\frac{tan\varphi }{cos\theta }\right)*R_{110}+\frac{\text{3}}{\text{2}}*\left(1-tan\theta \right)*R_{311}$

3) Analysis of interpolation results

Based on the characteristics of anisotropic etching rate, this study uses the cubic interpolation method (Cubic: Delaunay triangulation interpolation, which produces a smooth effect at local data points) and the double harmonic spline interpolation method(v4: Global interpolation, which produces high smoothing effects even with fewer global data points) in the non equidistant node binary interpolation function Griddata of MATLAB for interpolation calculation, the spherical mesh difference resolution (θ, φ) is (5, 5). According to Fig 1 (c), it can be seen that under the etching conditions of 70℃ + 40wt% KOH, the values of ${\text{R}}_{100}$=0.599um/min, ${\text{R}}_{110}=$1.15um/min，${\text{R}}_{111}=$0.01um/min and ${\text{R}}_{311}=$0.95um/min were calculated using the interpolation method for the etching rate of the entire crystal planes in this study. The following whole crystal plane etching rate curves and three-dimensional spherical coordinate velocity vector graphs were obtained.

**Fig 9:**
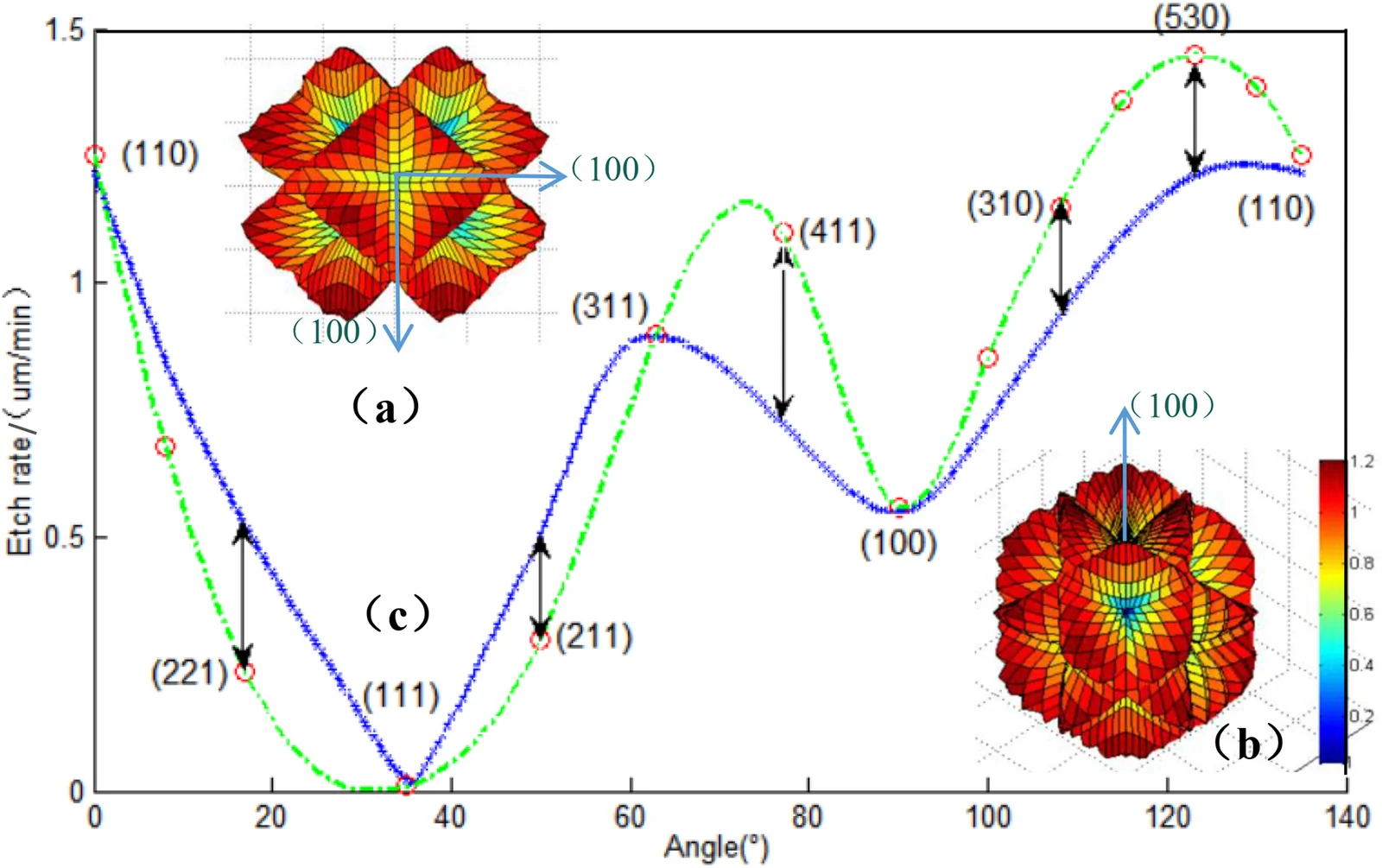
Interpolation results of the etching rate of all crystal planes under the condition of 70℃ 40% KOH: (a) Top view result presentation in <111 > orientation, (b) Top view result presentation in <100 > orientation, (c) comparison of the interpolation results（Green curve） with the experimental data(Blue curve).

The comparison between the interpolation results shown in Fig 9(in the uploaded file) and experimental data indicates that the etching rates for all crystal planes of silicon, derived from interpolations using the (111), (110), (100), and (311) sample crystal planes, generally align with the experimental curve trends. However, due to the limited number of crystal planes involved in the sampling calculations, the interpolation results in certain regions (e.g., (211), (411), and (530) regions) exhibit relatively low accuracy, with average errors exceeding 10%.

**Fig 10:**
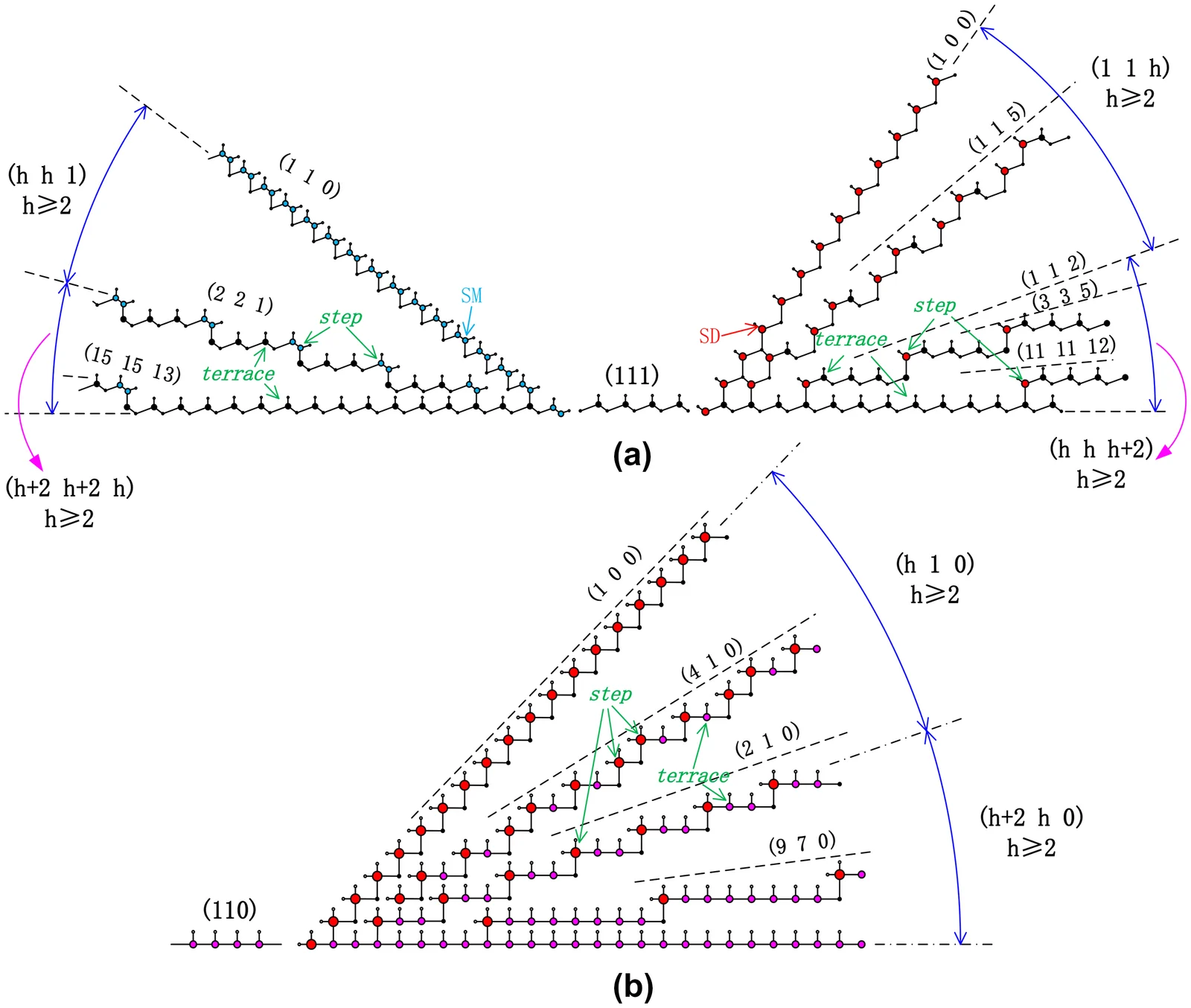
The {h,k,l}distribution diagram: (a) Top view of the (110) plane; (b) Top view of the (100) plane.

**Fig 11:**
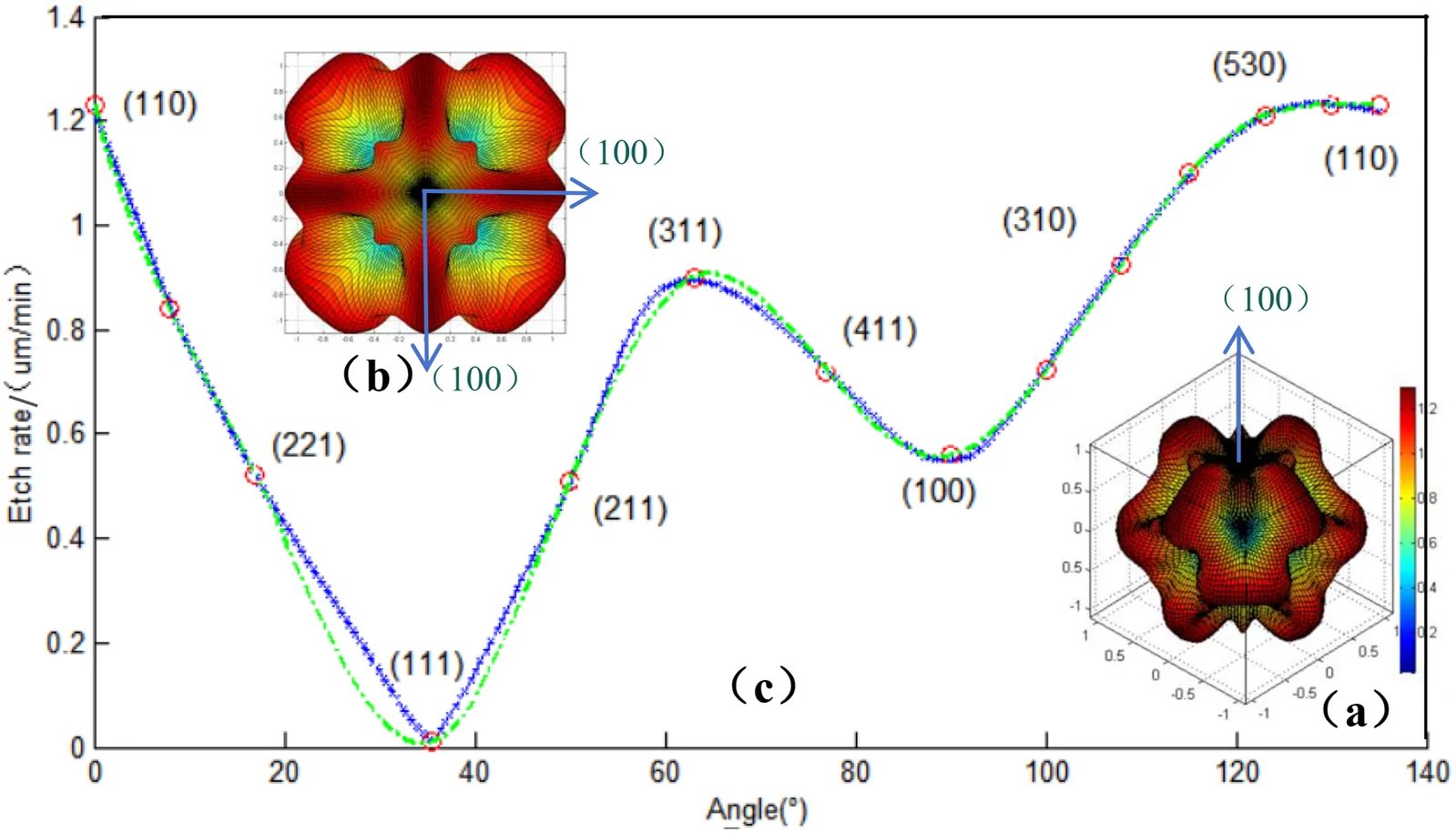
All the plane etching rates obtained by interpolation of the etching rates of the thirteen crystal planes: (a) Top view result presentation in <111 > orientation, (b) Top view result presentation in <100 > orientation, (c) comparison of the interpolation results（Green curve） with the experimental data (Blue curve).

The crystal planes of silicon can be categorized into three fundamental planes:(100), (110), and (111), and six crystal plane families: {h h h + 2}, {1 1 h}, {h + 2 h + 2 h}, {h h 1}, {h + 2 h 0}, and {h 1 0}. The surface atoms of the three fundamental planes exhibit a uniform atomic composition and lie at identical depths along the Z-axis, whereas the surface atoms of any plane within the six crystal plane families do not share the same depth, forming an alternating structure of steps and terraces. As shown in Fig 10(a)(in the uploaded file), the projection of the (110) plane reveals two crystal plane families between the (111) and (110) planes: {h h h + 2} and {1 1 h}, which are obtained by rotating the (111) plane around the (100) plane by a specific angle. Similarly, the two crystal plane families {h + 2 h + 2 h} and {h h 1} between the (111) and (110) planes can be derived through analogous rotation techniques. Likewise, the projection of the (100) plane in Fig 10(b)(in the uploaded file) demonstrates that two crystal plane families:{h + 2 h 0} and {h 1 0}, exist between the (110) and (100) planes, each obtained by rotating the (110) plane by corresponding angles relative to the (100) plane.The research team discovered that although different crystal faces within the same family exhibit differences in atomic structure, their anisotropic characteristics share similarities, with adjacent crystal faces demonstrating distinct transitional patterns in etching rates and morphology. To ensure computational accuracy and reduce computational burden during interpolation calculations, the study selected 13 sample crystal faces, comprising three fundamental faces plus six family faces, with priority given to those located at maximum or minimum points on the rate curves. For the four sample crystal faces shown in Fig 9, additional planes were included where significant rate interpolation errors were observed. Overall, thirteen sample crystal faces were chosen: (100), (110), (111), (311), (211), (411), (331), (221), (530), (540), (310), (210), and (320). When interpolating the etching rates of thirteen main crystal planes, the simulation rate is almost identical to the experimental rate, with an average error of less than 5% in the etching rate of each crystal plane, as shown in Fig 11(in the uploaded file). It can be seen that when the number of samples is sufficient, the level set interpolation method proposed in this study can effectively simulate the anisotropic etching characteristics of single crystal silicon.

4) 3D simulation

**Fig 12:**
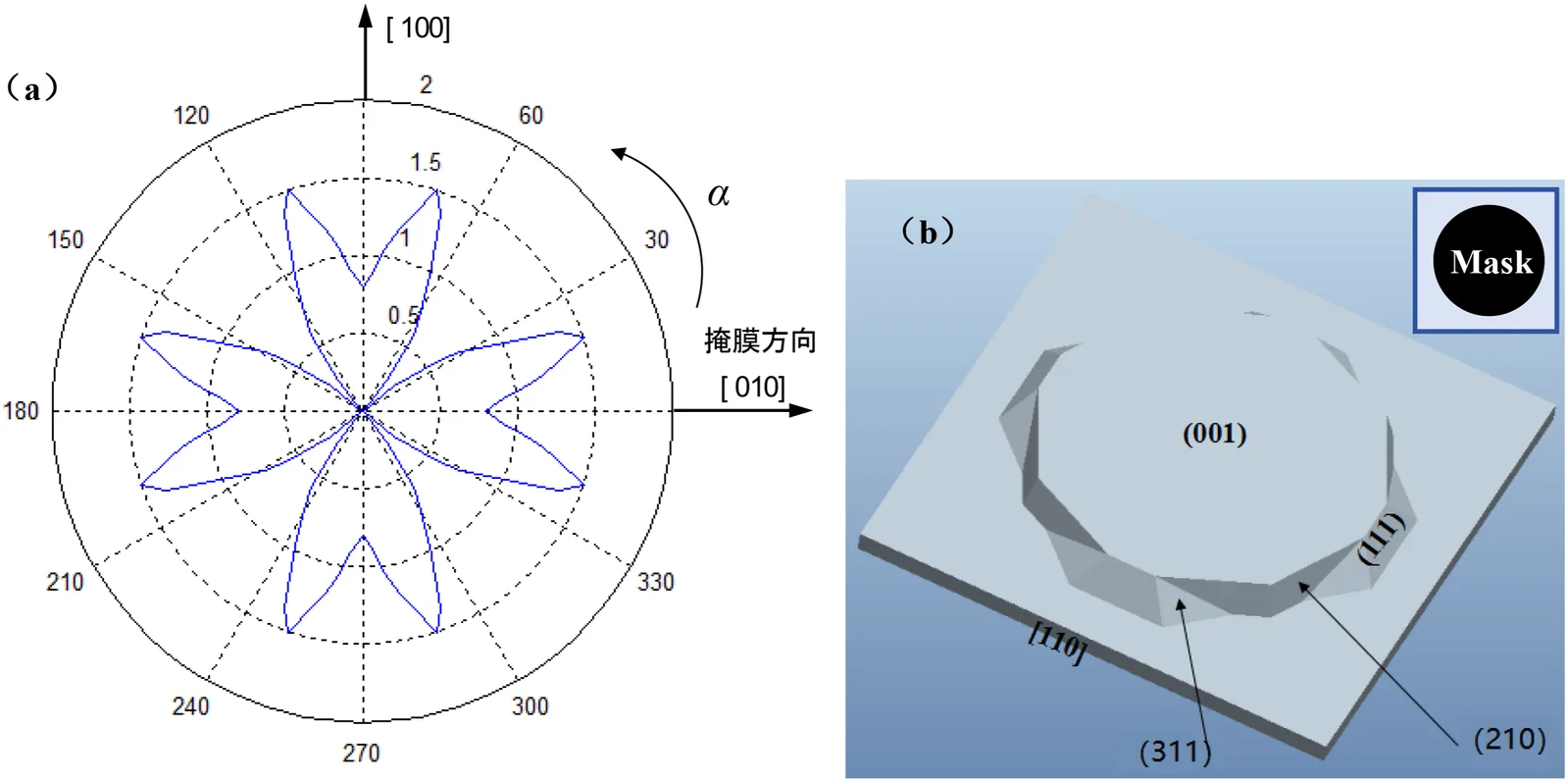
(a) Undercutting rate corresponding to different mask orientations; (b) Simulated result of circular mesa mask etching.

The established method for identifying etching profile at convex mask positions demonstrates that the maximum undercutting rate occurs at the mask’s convex angle. This establishes the corresponding crystal planes and undercutting rates for different mask orientations, as shown in Table 3. Fig 12(a)(in the uploaded file) reveals that when the mask orientation is [130], the profile undercutting rate reaches 1.56μm/min in 30wt% KOH, with the corresponding {210} matching the plane at the convex angle observed in Fig 12 (b)(in the uploaded file). These results confirm that the Level Set method accurately maps each gradient to a plane during etching, aligning with the etching profile evolution laws while maintaining high simulation precision.

**Table 3:** Etching rates of planes in 30wt% KOH with different mask orientations.

Mask direction	[110]	[450]	[230]	[350]	[120]	[250]	[130]	[140]	[150]	[160]	[010]
Corner α	pi/4	0.90	0.98	1.03	1.11	1.19	1.25	1.33	1.37	1.41	pi/2
Crystal plane	{111}	{455}	{233}	{355}	{122}	{255}	{210}	{140}	{150}	{160}	{010}
Undercut rate（um/min）	0.006	0.25	0.59	0.74	0.97	1.36	1.56	1.29	1.19	1.11	0.80

## 4. Analysis of the simulation results

### 4.1. Spherical silicon etching simulation

**Fig 13:**
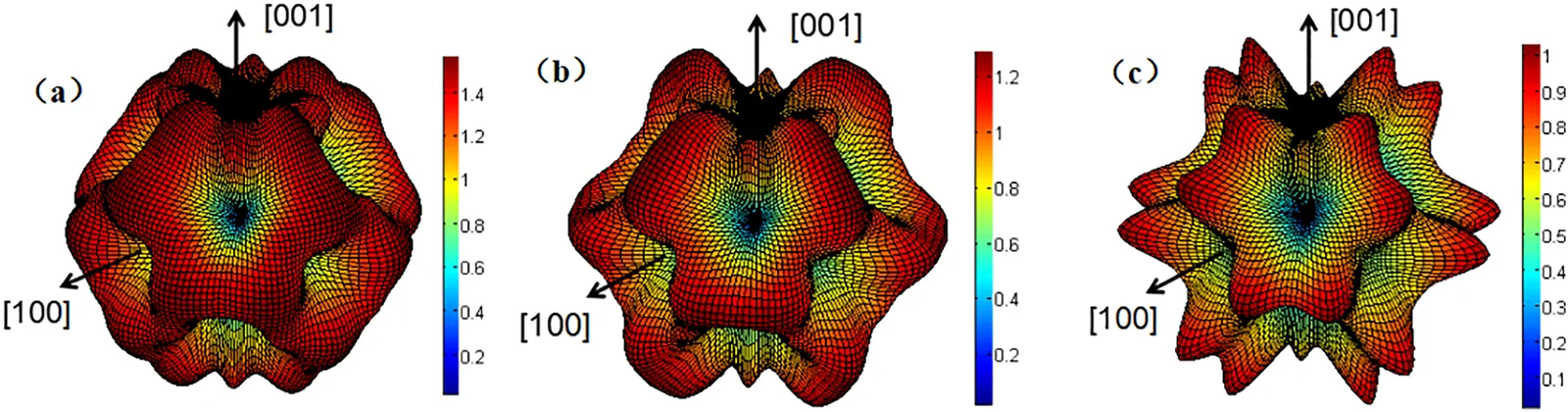
Interpolation etching rate map of silicon: (a) 70°C，30wt% KOH solution, (b) 70℃，40wt% KOH solution at, (c) 70°C，50wt% KOH solution.

Table 4 displays the etching rates of thirteen sample planes for silicon under three distinct etching conditions. Using the interpolation method proposed in this study, we obtained the following differential etching rate distribution across all crystal planes of silicon in the three etching solutions, along with corresponding simulation results for silicon spheres, as detailed below Fig 13(in the uploaded file):

**Table 4:** Etching rates of thirteen typical crystal planes.

Crystal Plane		100	110	210	211	221	310	311	320	331	530	540	111	411
Etching Rate μm/min	30wt% KOH	0.80	1.46	1.56	1.32	0.71	1.46	1.44	1.54	1.16	1.56	1.51	0.01	1.34
	40wt% KOH	0.60	1.15	1.26	0.75	0.54	1.00	0.95	1.10	0.80	1.18	1.13	0.01	0.78
	50wt% KOH	0.54	0.87	0.96	0.62	0.32	0.76	0.74	1.01	0.49	1.03	0.91	0.01	0.62

**Fig 14:**
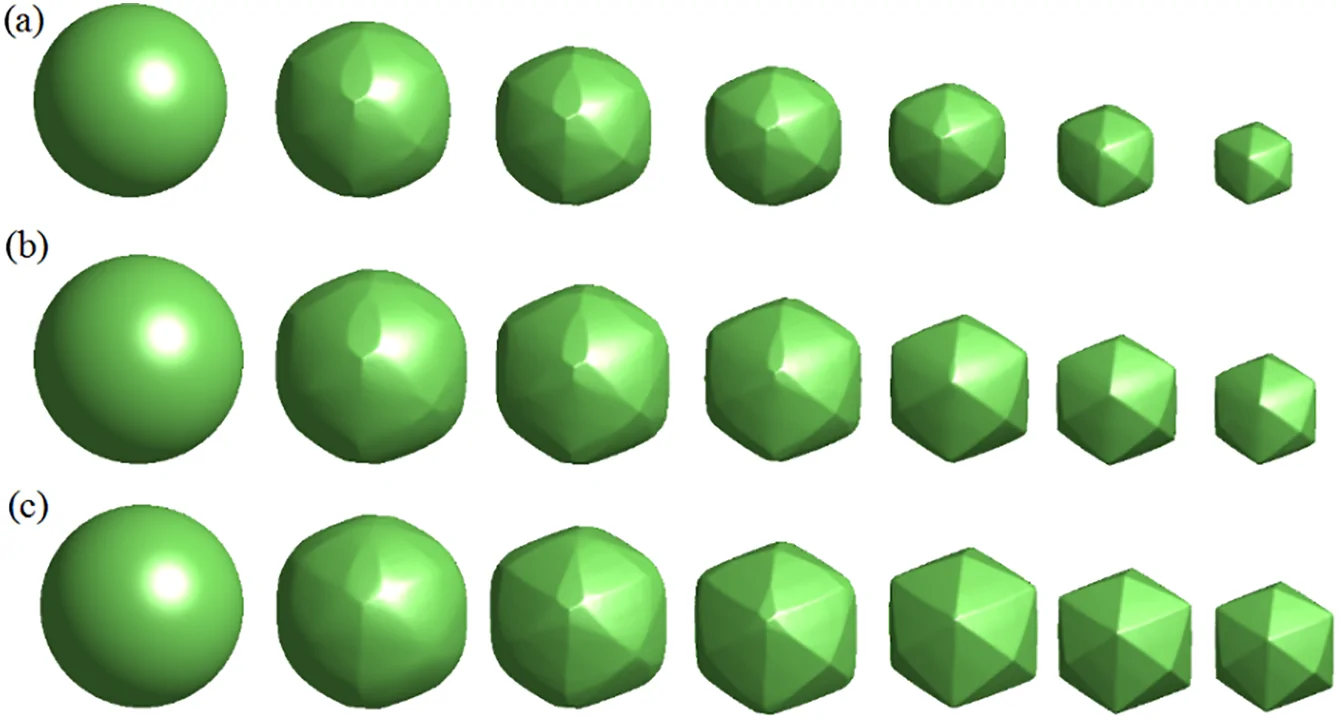
Simulation results of silicon sphere etching at different time points: (a) 70°C，30wt% KOH solution, (b) 70℃，40wt% KOH solution, (c) 70°C，50wt% KOH solution.

Figure 14(in the uploaded file) shows the simulation results of silicon spheres with a diameter of 100μm after being etched for 0 min, 5 min, 10 min, 15 min, 20 min, 25 min and 30 min in three different etching solutions. As shown in Fig 14, the etching profile of silicon spheres remains consistent despite the differing concentrations of the three KOH solutions. This indicates that the etching rate on the fast plane is largely uniform under three conditions, which aligns with the rate distribution of the 13 sample planes listed in Table 4. Additionally, it is important to note:as the concentration of the KOH solution increases, the etching rate of the silicon spheres shows a significant decrease.

### 4.2. Wafer etching simulation

1) Window mask

**Fig 15:**
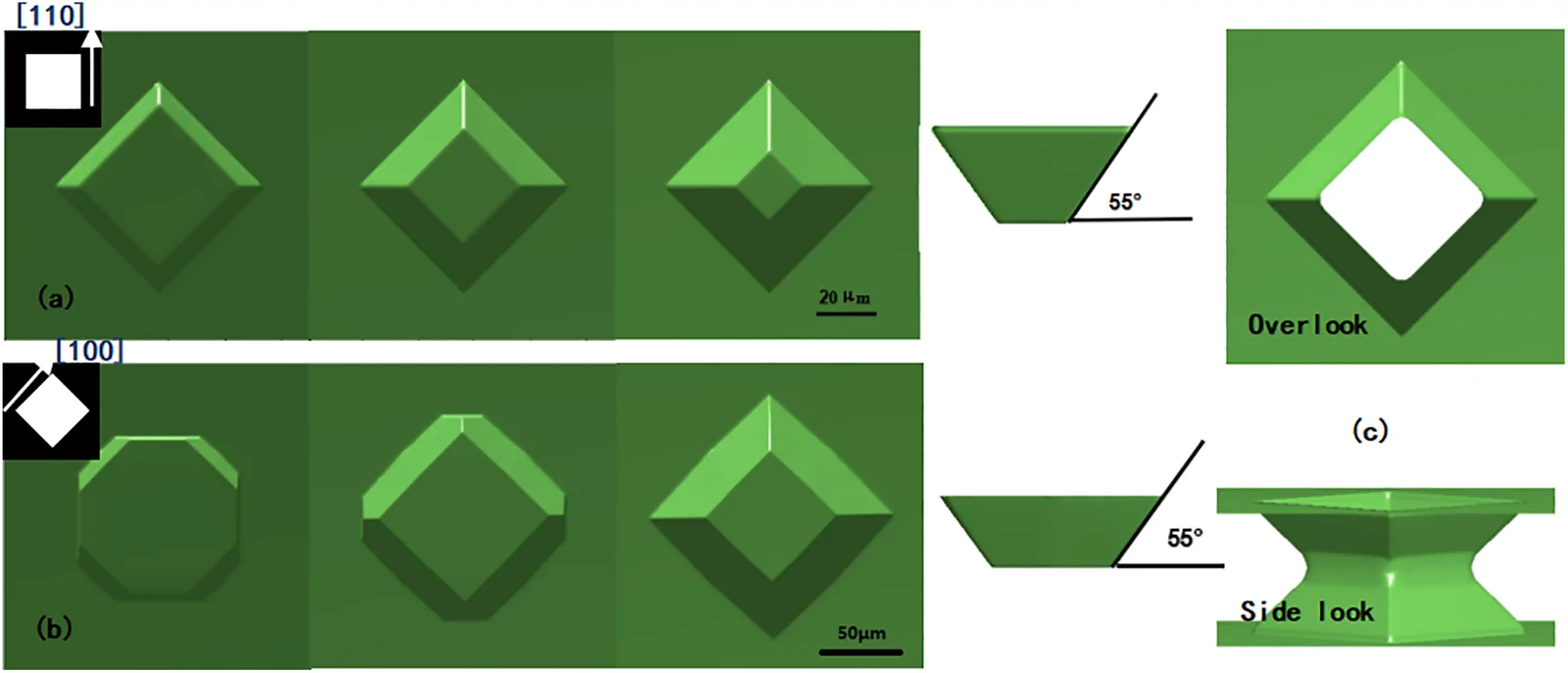
Simulation of window mask etching of Si(100) wafer in 70°C 30wt% KOH solution: (a) mask orientation [110], (b) mask orientation [100], (c) double-sided etching with mask orientation [110].

Figure 15(in the uploaded file) shows the simulation of Si (100) wafers (mask side-length 50um)etched for 10,20, and 30 minutes in a 70°C 30wt% KOH solution.When the mask orientation is [110], the final etched profile exhibits a 55°inclination angle with minimal undercutting,and the four profiles are {111}；When the mask orientation is [100], the initial profiles consists of{100}and{111}. As etching progresses, the{111}gradually dominates until the{100}completely disappears. At the end of etching, the final etched shapes in Fig 15 (a) and Fig 15 (b) are identical, but Fig 15(b) is significantly larger in size than Fig 15(a). Fig 15(c) shows the structural appearance of the wafer during double-sided etching with mask orientation [110]，and exhibits excellent agreement with experimental observations.

2) Mesa mask

**Fig 16:**
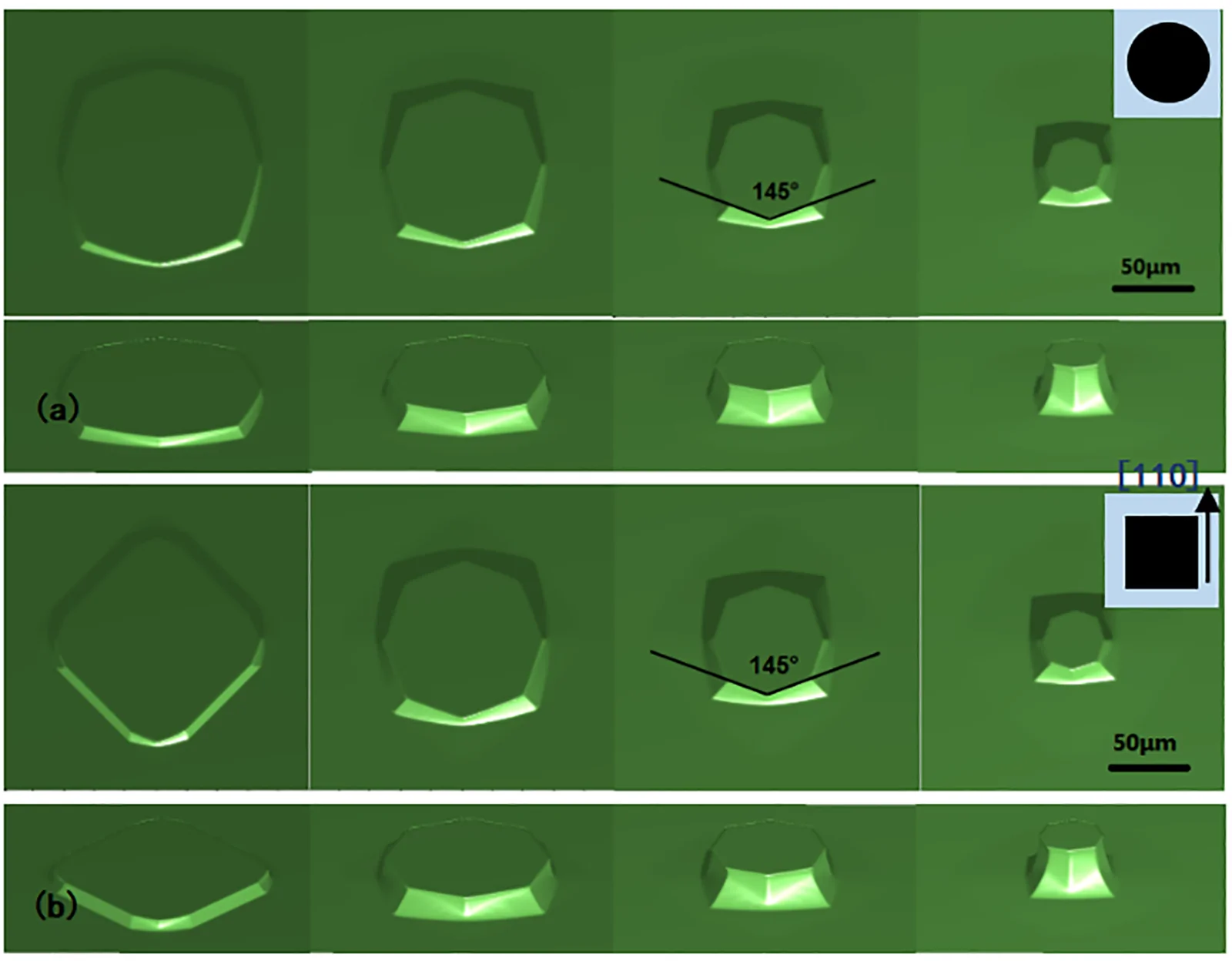
Simulation of mesa mask etching of Si(100) wafer in 70°C 30wt% KOH solution: (a) circular mesa mask, (b) mask orientation [100].

Fig 16 (in the uploaded file)shows the simulation of Si(100) wafers with circular mesa mask (diameter 140μm) and square mesa mask (side-length 100μm) after etching for 10,20,30, and 40 minutes in a 70°C 30wt% KOH solution.For the circular mesa mask depicted in Fig 16 (a), profile etching becomes apparent upon initiation, with the{210}exhibiting higher undercutting rates gradually dominating, and the projection angle of the adjacent sidewall is 145°.In contrast, the square mesa shown in Fig 16 (b) initially displays{111}sidewall at the mask edge. As etching progresses, the{210}with higher undercutting rates at the convex corner emerges and becomes dominant again, while the {111} progressively disappears.This demonstrates that, provided the etching time is sufficiently long, any mesa mask wafer will ultimately develop a similar sidewall structure.

3) Comparison of simulation and experimental results

**Fig 17:**
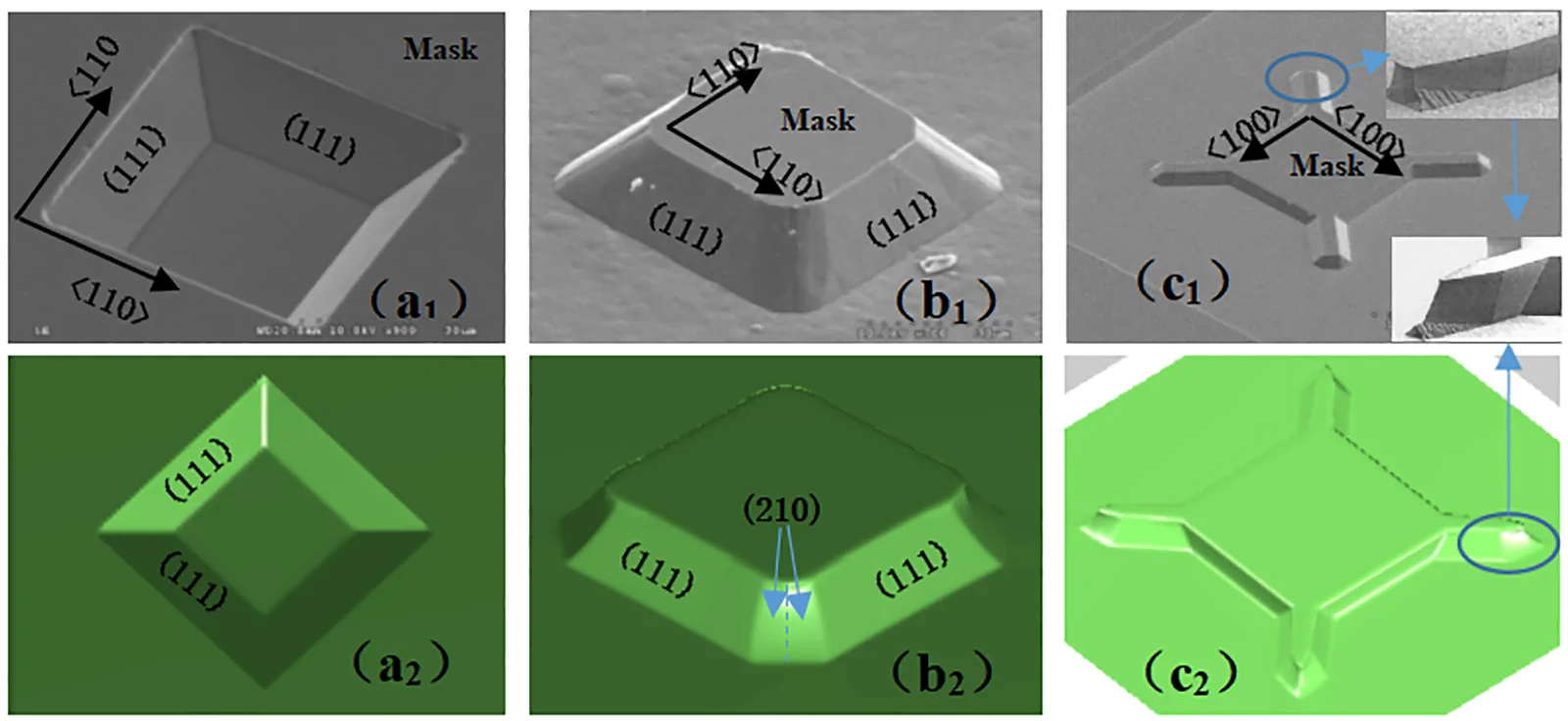
Comparison of experimental results (a1-c1) and simulation results (a2-c2) of mask etching: (a) window mask; (b) mesa mask; (c) Cross star composite mask.

Fig 17 (in the uploaded file)shows the comparative data of simulated profiles and experimental results of window mask, mesa mask, and composite mask etching under 70℃ 40wt% KOH etching conditions. As shown in Figure 17(a1), the etching structure of the Si (100) window mask is a truncated pyramid cavity, and all structural planes are relatively smooth and flat. Experimental measurements show that the four isosceles structural planes are Si(111) and the bottom plane is Si(100), whose angle of them is approximately 125.26°. The interpolation calculation simulation in Figure 17(a2) exhibits a highly consistency with the experimental results, both displaying truncated pyramid cavity composed of four (111) planes. Similarly,Figure 17(b1) shows a truncated pyramid with four smooth Si(111), and the edge of the convex corner of the mesa mask gradually becomes blunt after etching, forming an intersecting edge composed of two symmetrical sides. Further research has found that these two symmetrical sides are the (210) planes with the maximum etching rate shown in Fig 5(b). The simulation results shown in Figure 17 (b2) also exhibit a similar truncated pyramid, whose convex corner is composed of two (210) planes. In addition, the cross star composite mask shown in Figure 17(c1) forms a convex structure composed of four (100) profiles perpendicular to the edges and four wedge-shaped top angles after etching, and the wedge-shaped top angles are composed of two large angle symmetrical sidewall surfaces; the simulation results shown in Figure 17 (c2) also demonstrate similar side wall and top corner structures. Compared with Gosalvez et al.’s method of reconstructing the etch rate distribution of whole planes using three basic crystal planes and their proposed 3D etching model, this study achieves further improvements in both differential etch rates and 3D etching simulation results by incorporating more extreme-rate crystal planes into the differential calculation, with the average error remaining below 5%.

### 4.3. Application of level set method in other crystal etching

**Fig 18:**
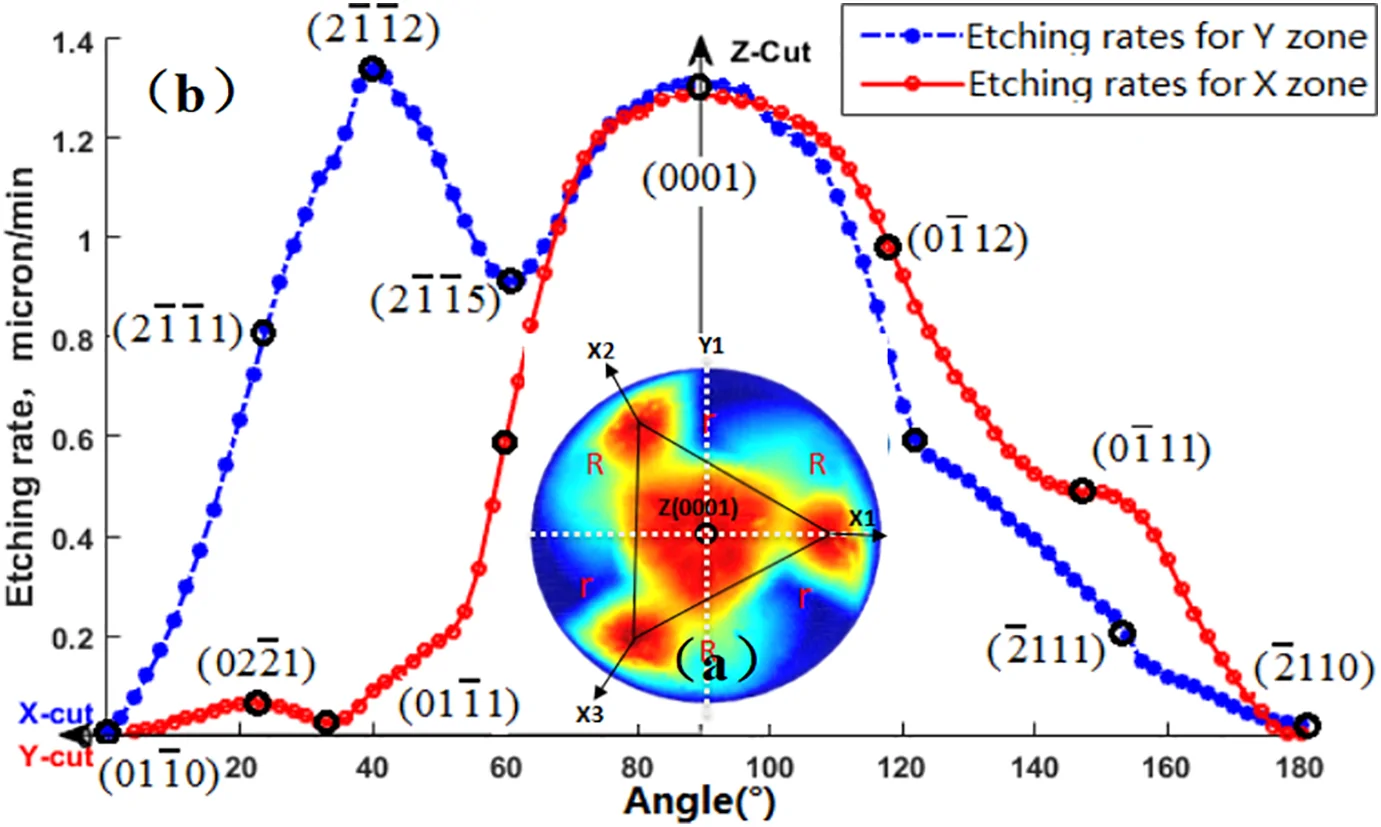
(a) Etch rate distribution in saturated NH4HF2 solution at 80 C for a hemisphere etched for 95 minutes. (b) Corresponding etch rates for the X and Y crystallographic zones.

**Fig 19:**
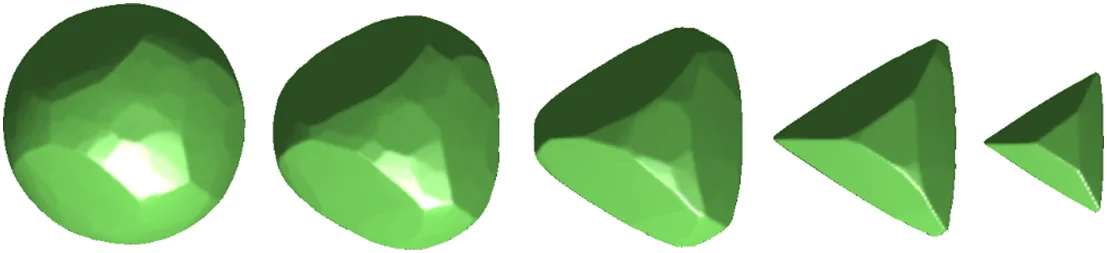
Simulation results of quartz sphere etching at different time points.

**Fig 20:**
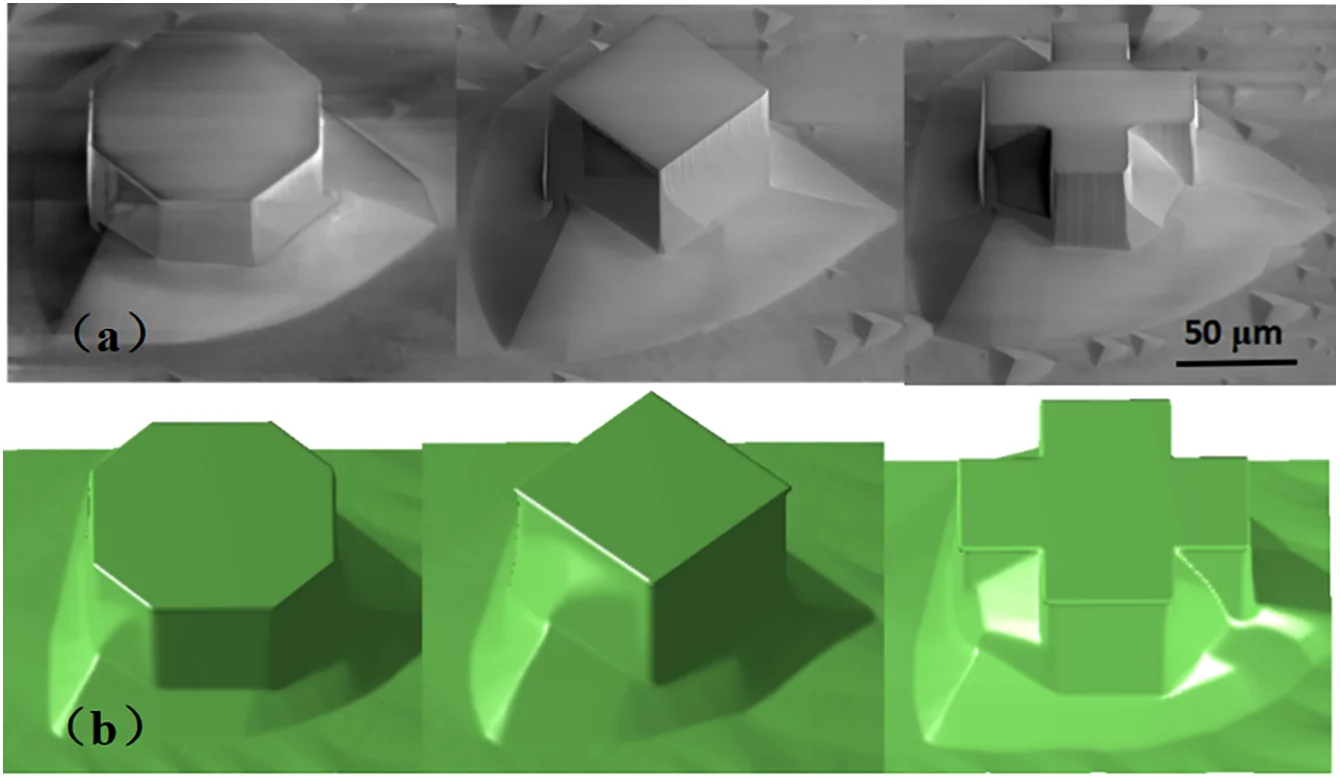
Comparison of experimental results (a) and simulation results (b) with different mask.

The Level Set method proposed in this study demonstrates high precision not only in simulating 3D etching structures of silicon but also excels in etching other crystal structures such as quartz. Fig 18(in the uploaded file) shows the etching rate distribution of a quartz sphere etched in 80°C saturated NH_4_F and HF mixed solution. Building upon this quartz etching rate distribution and the Level Set modeling method developed in this study, the constructed quartz etching simulation model also achieves high-precision etching simulations of arbitrary mask wafers under various etching conditions. Fig 19 (in the uploaded file)presents the etching simulation of quartz spheres, while Fig 20(in the uploaded file) compares experimental results with simulation results for Z-cut wafers with three different masks. It is evident that the quartz etching model based on Level Set possesses both process simulation capabilities and high-precision simulation performance.

## 5. Conclusion

This study systematically investigates the etching profile evolution mechanisms of anisotropic wet etching in silicon, and A simulation model based Level Set method was developed to predict 3D etching structures. Through hemispherical etching experiments, we obtained etching rates of some typical planes under different etching systems (KOH, TMAH). The etching rate interpolation model is constructed to reconstruct the etching rate of any crystal plane with a small number of sample planes. The level set motion interface control function and interface evolution equations were then established to dynamically track the profile evolution of etched structures. The simulation successfully reproduced 3D profile etched with various masks (windows, mesa) under multiple etching conditions. Furthermore, this method was successfully extended to quartz etching simulations, achieving results highly consistent with experimental data. The results show that the proposed method is suitable for predicting the anisotropic etching profiles of various crystal materials.

## Supporting information

S1 File Interpolation etching rate map of silicon, mask1_silicon, silicon sphere etching, window mask etching of Si(100) wafer 70°C 30wt% KOH, 30%KOH，Undercutting rate corresponding to different mask orientations, Silicon_30wt%KOH_70C (Interpolation etching), Silicon_40wt%KOH_70C (Interpolation etching), Silicon_50wt%KOH_70C (Interpolation etching), silicon_xoy_interpolation, V_3D_interpolation. (ZIP)
